# Organoaluminum Compounds as Catalysts for Monohydroboration of Carbodiimides

**DOI:** 10.1002/chem.201902000

**Published:** 2019-08-13

**Authors:** Qiumiao Shen, Xiaoli Ma, Wenling Li, Wenqing Liu, Yi Ding, Zhi Yang, Herbert W. Roesky

**Affiliations:** ^1^ School of Chemistry and Chemical Engineering Beijing Institute of Technology Beijing 100081 P. R. China; ^2^ Institute of Inorganic Chemistry Georg-August-Universität Göttingen Tammannstrasse 4 37077 Göttingen Germany

**Keywords:** carbodiimide, catalysts, hydroboration, organoaluminum

## Abstract

The effective catalytic activity of organoaluminum compounds for the monohydroboration of carbodiimides has been demonstrated. Two aluminum complexes, **2** and **3**, were synthesized and characterized. The efficient catalytic performances of four aluminum hydride complexes L^1^AlH_2_ (L^1^=HC(CMeNAr)_2_, Ar=2,6‐Et_2_C_6_H_3_; **1**), L^2^AlH_2_(NMe_3_) (L^2^=*o*‐C_6_H_4_F(CH=N‐Ar), Ar=2,6‐Et_2_C_6_H_3_; **2**), L^3^AlH (L^3^=2,6‐bis(1‐methylethyl)‐*N*‐(2‐pyridinylmethylene)phenylamine; **3**), and L^4^AlH(NMe_3_) (L^4^=*o*‐C_6_H_4_(*N*‐Dipp)(CH=N‐Dipp), Dipp=2,6‐*i*Pr_2_C_6_H_3_; **4**), and an aluminum alkyl complex L^1^AlMe_2_ (**5**) were used for the monohydroboration of carbodiimides investigated under solvent‐free and mild conditions. Compounds **1**–**3** and **5** can produce monohydroborated *N*‐borylformamidine, whereas **4** can afford the *C*‐borylformamidine product. A suggested mechanism of this reaction was explored, and the aluminum formamidinate compound **6** was characterized by single‐crystal X‐ray, also a stoichiometric reaction was investigated.

## Introduction

Main‐group metal catalysts are considered as a commercially useful and environmentally friendly class of compounds, especially when compared with most of the transition metals. Their application and catalytic properties have attracted much attention.^1^ In particular, the increasing desire to investigate the use of earth‐abundant metals has motivated researchers to invest considerable scientific efforts in aluminum complexes, owing to their inherent low toxicity and the ubiquitous availability of the element aluminum.^2^

In the past few years, rapid advances in the application of organoaluminum complexes have been witnessed. These complexes have been employed as catalysts in hydrogenation dehydrocoupling, hydroboration, and hydrosilylation reactions.^3^ The organoboron compounds derived from hydroboration reactions are crucial organic intermediates in various chemical transformations and material synthesis.^4^ In recent years, the ability of organoaluminum complexes to work as efficient catalysts for the hydroboration of unsaturated bonds with C=E (E=O,^5^ C,^6^ N^7^), and C≡E motifs (E=C,3a, 6b N6a, 8) was discovered. Intense work was invested in the hydroboration of unsaturated bonds through the use of aluminum‐containing pre‐catalysts. Moreover, organoaluminum compounds have been generally applied in hydroamination reactions.^9^ Even the reduction of CO_2_ was explored by using organic alkaline earth metal and organic transition‐metal catalysts.^10^

However, in those main‐group metal‐catalyzed reactions, the skeleton of the unsaturated compound contains only one unsaturated bond, such as an organic imine. Studies of the catalytic hydroboration of heterocumulenes using main‐group element‐based pre‐catalysts are limited. To the best of our knowledge, an aluminum complex as catalyst for the hydroboration of isocyanates and carbodiimides (E=C=E′; E=RN, RRC; E′=R′N, O, S) has not been reported.

Carbodiimides with N=C=N skeletons,^11^ containing a chain of two double bonds, are commercially available and widely used in the synthesis of esters, guanidines, and isoureas.^12^ An aluminum‐catalyzed exploration of the hydroboration of carbodiimides is therefore very important.

**Scheme 1:**
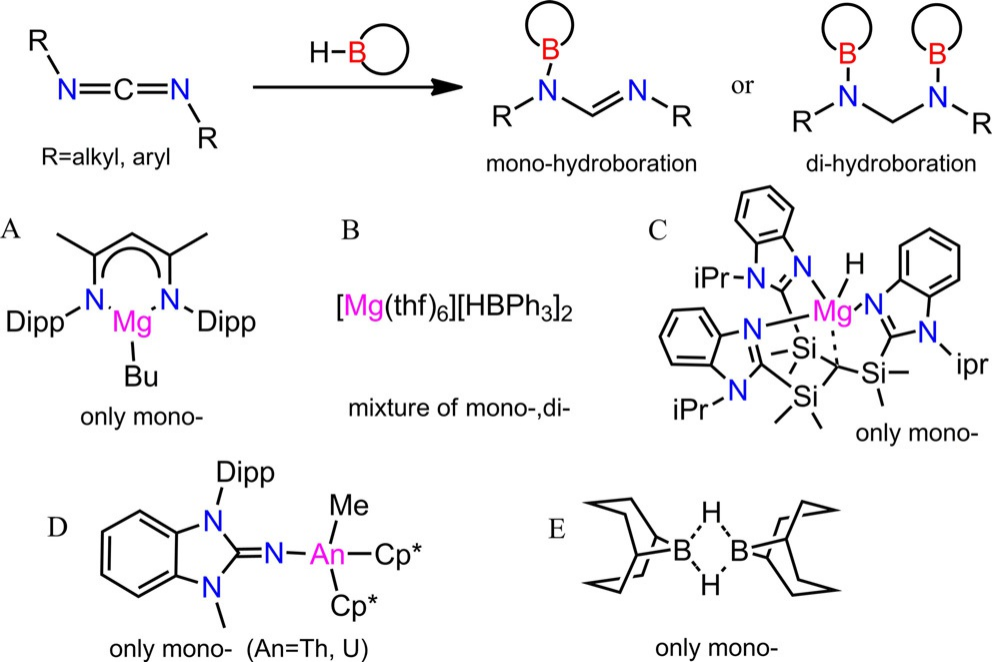
Overview of hydroboration with carbodiimides and catalysts. Dipp=2,6‐*i*PrC_6_H_3_, Cp*=1,2,3,4,5‐pentamethylcyclopentadiene.

The reaction of 9‐borabicyclo[3.3.1]nonane (9‐BBN) with carbodiimides at high temperature (160 °C) without catalyst,^13^ resulted in mixtures of mono‐ and bis‐hydroboration products without any selectivity. In 2016, a β‐diketiminato magnesium alkyl complex LMg*n*Bu (L=HC(CMeNAr)_2_, Ar=2,6‐*i*Pr_2_C_6_H_3_) was applied for the hydroboration of carbodiimide substrates, affording the monohydroborated product (Scheme 1 A).^14^ Furthermore, [Mg(thf)_6_][HBPh_3_]_2_ acts as a catalyst in carbodiimide hydroboration, resulting in a mixture of mono‐ and dihydroboration products in the presence of 1 equivalent of pinacolborane (HBpin), whereas bis(*N*‐boryl)aminal was only formed as the reduced product in the presence of 2 equivalents of Hbpin (Scheme 1 B).10c After that, [Tism^*i*PrBenz^]MgH was reported as a more active catalyst in the monohydroboration of two kinds of carbodiimides at room temperature (Tism^*i*PrBenz^=tris[(1‐ isopropylbenzimidazol‐2‐yl)dimethylsilyl)]methyl ligand; Scheme 1 C).^15^ Last year, eight different organoactinide complexes were shown to have good selectivity as catalysts for the hydroboration of carbodiimides with HBpin. All these reactions resulted in the monohydroborated product even in the presence of 2 equivalents of HBpin (Scheme 1 D).^16^ Very recently, the commercially available hydroborane (H‐BBN)_2_ was reported as a more efficient metal‐free catalyst for the monohydroboration of carbodiimides (Scheme 1 E).^17^

**Scheme 2:**
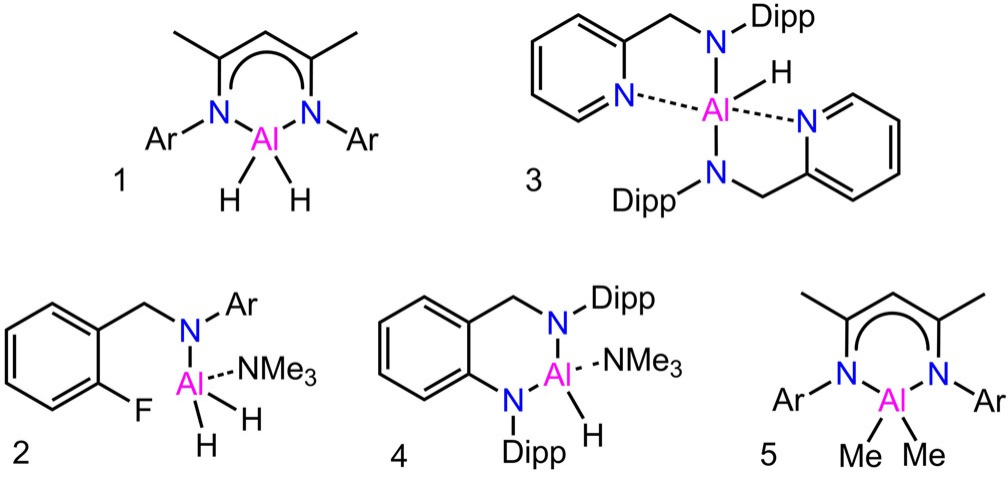
Compounds **1**–**5**. Ar=2,6‐Et_2_C_6_H_3_, Dipp=2,6‐*i*PrC_6_H_3_.

Herein, we report an unprecedented organoaluminum‐catalyzed highly efficient and highly selective monohydroboration process of commercially available carbodiimide substrates with pinacolborane (HBpin) under mild and solvent‐free reaction conditions. Five different aluminum complexes (**1**–**5**) including aluminum hydride and aluminum alkyls were used to explore their catalytic properties (Scheme 2).

## Results and Discussion

The aluminum hydride L^1^AlH_2_ (**1**; L^1^=HC(CMeNAr)_2_, Ar=2,6‐Et_2_C_6_H_3_),^18^ used as a highly active catalyst for the hydroboration of aldehydes and ketones before,5b was selected as a target for potential catalysis in this research. To explore the catalytic influence of different organoaluminum compounds supported by β‐diketiminato ligands or Schiff base ligands, compound **2** was prepared from reaction of L^2^ (*o*‐C_6_H_4_F(CH=N‐Ar), Ar=2,6‐Et_2_C_6_H_3_)^19^ with equimolar H_3_Al**⋅**NMe_3_ in toluene at 0 °C, and compound **3** was prepared from reaction of L^3^ (2,6‐bis(1‐methylethyl)‐*N*‐(2‐pyridinylmethylene)phenylamine)^20^ with equimolar H_3_Al**⋅**NMe_3_ in toluene at 0 °C. When adjusting the ratio of L^3^ and H_3_Al**⋅**NMe_3_ to 1:1 or 2:1, only the dimerization product was obtained. Both complexes were characterized by single‐crystal X‐ray diffraction analysis. L^4^AlH(NMe_3_) (**4**; L^4^=*o*‐C_6_H_4_(*N*‐Dipp)(CH=N‐Dipp), Dipp=2,6‐*i*Pr_2_C_6_H_3_) and the aluminum alkyl compound L^1^AlMe_2_ (**5**) were prepared according to literature methods.^18^, ^19^, ^21^

**Figure 1:**
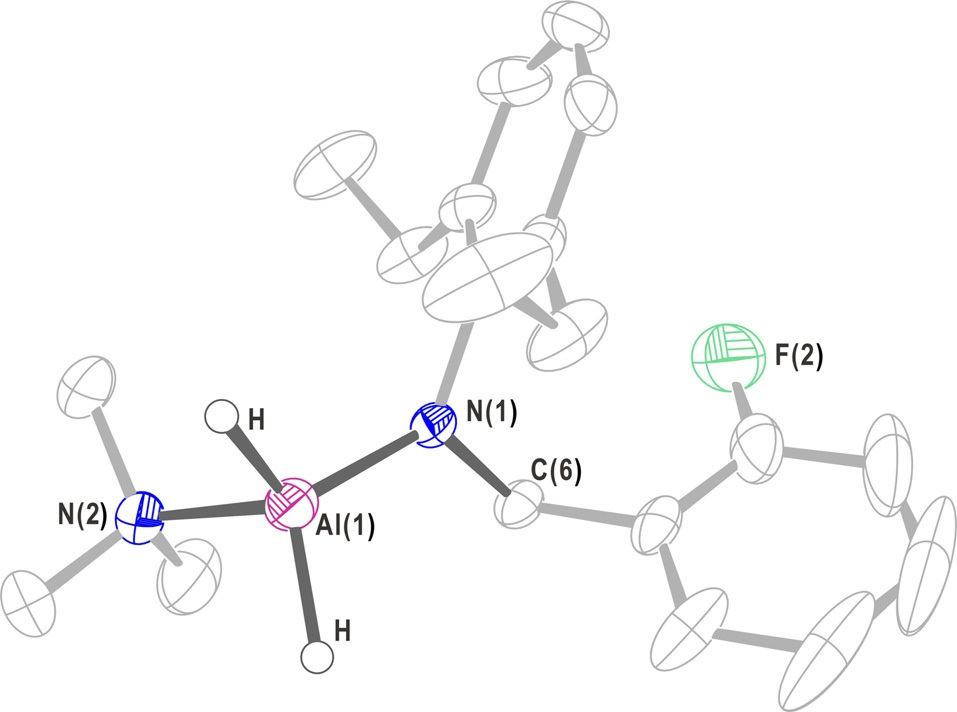
X‐ray single‐crystal structure of **2**. Anisotropic displacement parameters are depicted at the 50 % probability level. The hydrogen atoms are omitted for clarity, except for that of the Al−H bond. Selected bond lengths (Å) and angles (deg): Al(1)−N(1) 1.8152(13), Al(1)−N(2) 2.009(2), N(1)−C(6) 1.4690(18); N(1)‐Al(1)‐N(2) 110.14(8), C(6)‐N(1)‐Al(1) 119.92(10).

X‐ray diffraction analysis quality single crystals of **2** were obtained in hexane (Figure 1). The obtained crystal belongs to the monoclinic system, the *P*2_1_/*c* space group. The lengths of the Al−N bond and the C(H_2_)−N bond in the compound are, respectively, consistent with the corresponding bond lengths in the previously reported compound [*o*‐C_6_H_4_F(CH_2_N‐ Dipp)]AlH_2_(NMe_3_).19a The sum of the bond angles around the Al center is 345.2°, indicating a quasi‐tetrahedral configuration. The bond length of Al(1)−N(2) in compound **2** is 1.8152(13) Å, which is in the range of a covalent bond. The Al(1)−N(2) bond length is 2.009(2) Å, which shows a coordinate bond. The bond length of N(1)−C(6) is 1.4690(18) Å, which is in the range of a single bond. This result also indicates that a Markov addition occurs between H_3_Al**⋅**NMe_3_ and the organic ligand L^2^.

**Figure 2:**
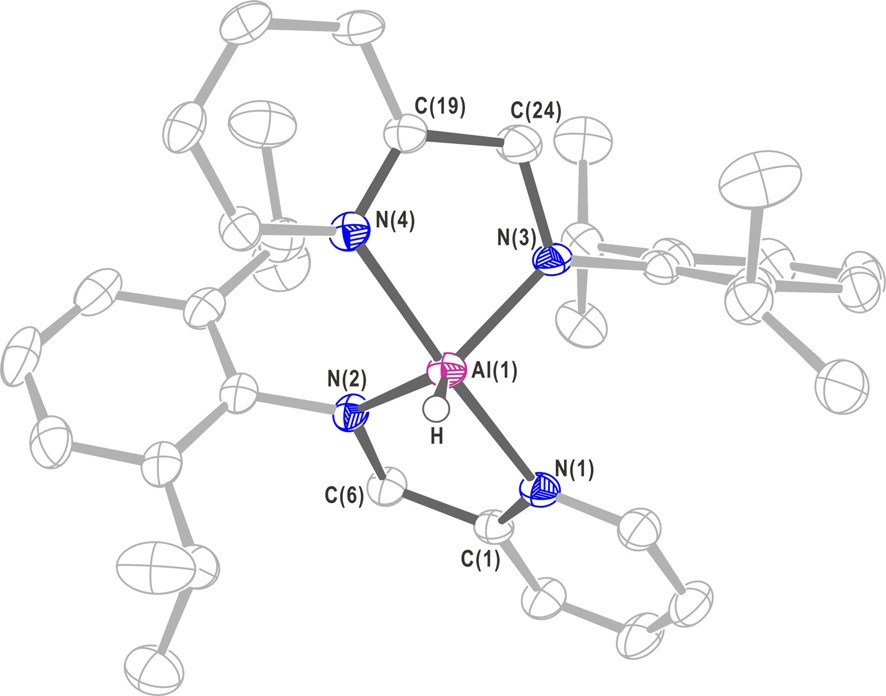
X‐ray single‐crystal structure of **3**. Anisotropic displacement parameters are depicted at the 50 % probability level. The hydrogen atoms are omitted for clarity, except for that of the Al−H bond. Selected bond lengths (Å) and angles (deg): Al(1)−N(1) 2.067(2), Al(1)−N(2) 1.860(2), Al(1)−N(3) 1.863(2), Al(1)−N(4) 2.090(2), N(2)−C(6) 1.450(3), N(3)−C(24) 1.448(4); N(2)‐Al(1)‐N(3) 124.35(11), N(1)‐Al(1)‐N(4) 171.24(10), N(3)‐Al(1)‐N(4) 81.40(8), N(2)‐Al(1)‐N(1) 81.27(8).

The single crystals of **3** for X‐ray diffraction analysis were obtained from a concentrated toluene solution (Figure 2), which belongs to the triclinic system, *P* space group. The bond length of Al(1)−N(2) is 1.860(2) Å and Al(1)−N(3) is 1.863(2) Å, which are consistent with that in [(DME)_3_Na][(IP^2−^)_2_Al] reported in the literature.20c The distance between the aluminum atom and the N atom in the pyridine ring, Al(1)−N(1), is 2.067(2) Å, and Al(1)−N(4) is 2.090(2) Å. Compared with those in reported compounds [(IP)AlCl_3_] and (IP^−^)_2_Al(OH), the bond lengths are similar to the Al−N coordinate bonds.20c, 22 Therefore, the bond between the Al atom and the N atom of pyridine in compound **3** is a coordinate bond. The sum of the bond angles at the center of the Al atom is 360.05°, which shows a quasi‐planar structure. It is worth noting that the bond angle of N(1)‐Al(1)‐N(4) is 171.24(10)°, and those two N atoms on the pyridine rings are close to being in a straight line with the Al atom. This indicates that the center of the aluminum atom exhibits an irregular double‐cone configuration.

At the beginning of this investigation, the catalytic hydroboration of 1 equivalent of 1,3‐bis(2,6‐diisopropylphenyl) (DippNCNDipp) with 1.1 equivalent of pinacolborane (HBpin) at 70 °C in the presence of catalyst **1** (4 mol %) under neat conditions was carried out. To our surprise, the liquid precursors turned within 4 h to a solid with 96 % conversion of DippNCNDipp. Only the monohydroboration product *N*‐{B(OCMe_2_)_2_}‐2,6‐diisopropylphenylformamidinate (**d**) was obtained, without the formation of any byproduct (Table 1, entry 8).

**Table 1:** Optimization for the monohydroboration of different carbodiimides by using aluminum complex **1** as the catalyst.

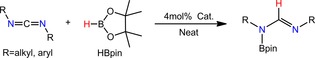					
Entry^[a]^	RNCNR	*t* [h]	*T* [°C]	Yield [%]^[b]^	Prod.
1	*i*Pr	12	70	79	**a**
2			80	96	
3^[c]^				99	
					
4	Cy	12	70	81	**b**
5			80	97	
					
6	*t*Bu	60	70	34	**c**
7			80	69	
					
8	2,6‐*i*Pr_2_C_6_H_3_	4	70	96	**d**
9		2	80	99	
10^[c]^				99	

[a] Reaction conditions: 0.04 mmol catalyst **1**, 1.1 mmol HBpin, 1 mmol carbodiimide. [b] By ^1^H NMR analysis. [c] With 2.1 mmol of HBpin.

The success of this initial test reaction encouraged us to expand the substrate scope to other commercially available carbodiimides. Subsequent hydroboration reactions with representative results are summarized in Table 1. In each case, the yield of the hydroborated product was calculated from the ratio of starting material and the target product by ^1^H NMR spectroscopy. Anisole (PhOMe) was used as an internal standard. Aliphatic carbodiimide *N*,*N*′‐diisopropylcarbodiimide (DIC) and *N*,*N*′‐dicyclohexylcarbodiimide (CyNCNCy) were investigated at 70 °C, affording the desired monohydroboration products **a** and **b** in 12 h with yields of 79 % and 81 %, respectively (Table 1, entries 1 and 4). When the steric bulk of the R group was increased to *tert*‐butyl (Table 1, entry 6) as the substrate, the hydroboration reaction occurred much slower.

To increase the yield and reduce the reaction time, a series of similar reactions were conducted under a slightly elevated temperature of 80 °C. The results are summarized in Table 1. The hydroboration of dialkylcarbodiimides resulted in a higher yield within the same reaction time. DIC and CyNCNCy achieved yields of 96 % and 97 %, respectively (Table 1, entries 2 and 5). In addition, the hydroboration of DippNCNDipp provided efficient turnover to the desired borylated products under 80 °C, achieving a yield of over 99 % within 2 h (Table 1, entry 9). Catalyst **1** functions more effective for DippNCNDipp. The results of reactions with DIC and CyNCNCy are quite similar. Furthermore, dialkylcarbodiimides with higher steric bulk resulted in longer reaction times.

Moreover, the catalytic hydroboration between 2.1 equivalents of HBpin with 1 equivalent of DippNCNDipp or DIC at 80 °C was carried out. In these reactions, only the monohydroboration products were detected (Table 1, entries 3 and 10). This indicated the lower reactivity of the remaining imine functionality, which is resistant to any further reduction, regardless of whether or not there is an excess of HBpin.

To compare the catalytic performances of the well‐defined aluminum complexes in this catalysis system, the scope was broadened. Catalytic aluminum hydride compounds **2**, **3**, and a reported pre‐catalyst aluminum alkyl compound **5** were used for the hydroboration of carbodiimides. Based on the results mentioned above, to ensure maximum efficiency and selectivity, 1.1 equivalents of HBpin were used in neat conditions at 80 °C for these reactions with a loading of 4 mol % catalyst. All representative results for the hydroboration of different types of carbodiimide substrates are listed in Table 2.

**Table 2:** Catalytic trials for the monohydroboration of different carbodiimides by using aluminum complexes **2** and **3** as catalysts and **5** as a pre‐catalyst.

Entry^[a]^	Cat.	RNCNR	*t* [h]	Yield [%]^[b]^
1	**2**	*i*Pr	24	99
2		Cy	24	95
3		*t*Bu	40	96
4		2,6‐*i*Pr_2_C_6_H_3_	48	95
				
5	**3**	*i*Pr	24	98
6		Cy	24	99
7		*t*Bu	60	31
8		2,6‐*i*Pr_2_C_6_H_3_	36	44
				
9	**5**	*i*Pr	24	93
10		Cy	24	91
11		*t*Bu	40	73
12		2,6‐*i*Pr_2_C_6_H_3_	48	77

[a] Reaction conditions: 0.04 mmol catalyst, 1.1 mmol HBpin, 1 mmol carbodiimide, at 80 °C. [b] By ^1^H NMR analysis.

Under optimized conditions, the catalytic hydroboration was investigated in the presence of compound **2**. It proceeded effectively to give the singly reduced amidinate compound without detection of any dihydroboration products. Although it took longer than that with compound **1**, both DIC and CyNCNCy were almost fully converted (Table 2, entries 1 and 2). It is worth mentioning that compound **2** performs better than compound **1** in the hydroboration of *t*BuNCN*t*Bu. It achieved a yield of 96 % in 40 h, which is much better than that of **1** (Table 2, entry 3). Compound **3** also worked very well as a catalyst for the hydroboration of DIC and CyNCNCy, affording the sole hydroboration product with yields of 98 % and 99 % (Table 2, entries 5 and 6), respectively, whereas the hydroboration of *t*BuNCN*t*Bu afforded only a yield of 31 % in 60 h. The yield of compound **3** catalyzed hydroboration of DippNCNDipp was 44 % (Table 2, entry 8) and did not increase further when the time was extended.

Compounds **2** and **3** showed quite different effects compared with compound **1** in the catalytic reaction between dialkylcarbodiimides and DippNCNDipp, respectively. Both take a long time for DippNCNDipp to be completely transformed. These results indicate that β‐diketiminato ligands and Schiff base ligands have a great influence on the catalytic effect. Those two kinds of ligands give different reaction times and yields under otherwise identical conditions. The ligand of compound **1** forms a conjugate six‐membered ring with aluminum, making the coordinate Al−N bond similar in length to the sigma Al−N bond, and the Al center shows Lewis acidity in compound **1**. The steric hindrance of compound **2** is smaller than that of compound **1**. However, the NMe_3_ group attached to aluminum might reduce the Lewis acidity of Al, owing to its electron‐donating effect, so its catalytic effect is not as good as that of compound **1**. In the organic skeleton of compound **3**, N belongs to pyridine and acts as a Lewis base. The Lewis basic pyridine of compound **3** provides two lone pairs of electrons to the Al atom through two coordinate Al−N bonds, which reduces the Lewis acidity of the aluminum. The aluminum center of compound **3** is sterically crowded, owing to the presence of two *N*‐Dipp groups of **3** in *trans* positions. All these factors make it more difficult for the Al atom to bind with one N at the carbodiimide. Above all, we presume that the catalytic performance might be improved by increasing the Lewis acidity of Al and reducing the steric hindrance. The hydroboration of *t*BuNCN*t*Bu is affected by the steric hindrance of the catalyst, where the spatial effect of the *t*Bu group dominates.

In the experiment catalyzed by the alkyl aluminum compound **5** (Table 2, entries 9–12), it was found that the catalytic effect of **5** is not as good as that of aluminum hydride. This drastic decrease in the catalytic effect may be due to the more stable Al−Me bond compared with the Al−H bond. Also, the methyl group acts as an electron‐donating group and is directly attached to aluminum, which reduces the Lewis acidity of Al. The steric hindrance of a methyl group is greater than that of a H atom, which prevents the bonding of the aluminum to HBpin and carbodiimide.

It can be observed that the catalytic effect is mainly influenced by the steric hindrance of the organic substituents on the carbodiimide. For example, the steric hindrance of the substituents of DippNCNDipp is larger than that of *t*BuNCN*t*Bu, but the phenyl group exhibits a planar structure, whereas the *t*Bu group exhibits a tetrahedral structure. The N of DippNCNDipp might be more likely approached by the catalyst or borane from the upper and lower directions, compared with the N of *t*BuNCN*t*Bu. Moreover, the electron density of the benzene ring is increased owing to the electron‐donating effect of the isopropyl group on the benzene ring. The conjugate effect of benzene with N=C=N increases the Lewis basicity of N. The reason why the hydroboration of DippNCNDipp is better than the hydroboration of dialkylcarbodiimides may be due to these aspects. This phenomenon also could be observed in the Mg‐catalyzed carbodiimide hydroboration reaction.^14^

**Scheme 3:**
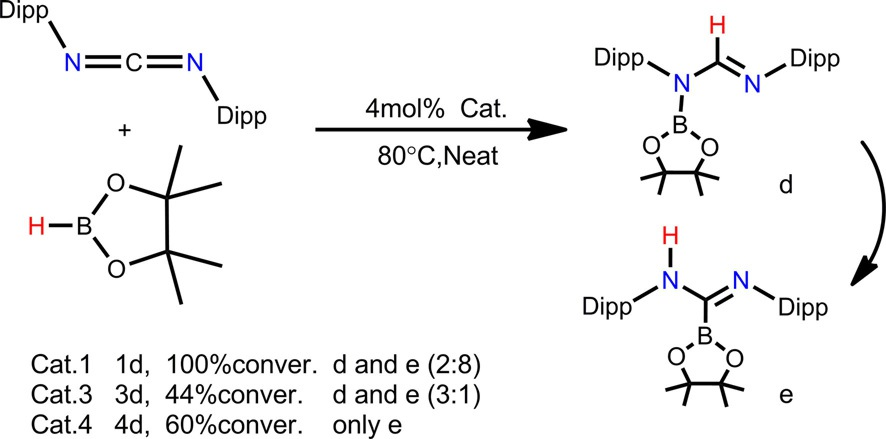
The conversion of product **d** to **e**.

During our attempt to increase the yield of compound **3** catalyzed hydroboration of DippNCNDipp by increasing the reaction time, we serendipitously discovered the formation of monohydroborated *C*‐borylformamidine product (**e**), which has never been reported before as far as we know (Scheme 3). It can be easily identified by the ^1^H NMR spectrum. The product **e** displayed a significant downfield characteristic resonance at *δ*=9.73 ppm (Ar‐N*H*‐C), which is quite different from the singlet resonance of **d** at *δ*=8.05 ppm of the formamidine (NC*H*N). If the reaction time was extended to 12 h at 80 °C, when using compound **1** as catalyst for the hydroboration of DippNCNDipp, a small amount of product **e** was also formed. It was discovered that after reaching the maximum conversion, the content of **e** increases, whereas **d** decreases with the extension of heating time, and thus **d** is slowly transformed into **e**. Then, we used compound **4** as the catalyst in the hydroboration of DippNCNDipp at 80 °C for 4 days. The highest conversion rate of 60 % can be obtained within 4 h, and finally only product **e** was obtained without any byproduct. Owing to the prolonged heating, product **d** tends to rearrange into a more stable form. Both products **d** and **e** are imines, there may be an intermolecular hydrogen bonding force formed by the H of C=NH of the product **e**, which makes **e** more stable than **d**. The electron density on the C=N bond of the imine **d** is larger than that on the C−N bond. The electrophilicity of boron gives Bpin a tendency to shift to the double bond to form a B−C bond.

**Figure 3:**
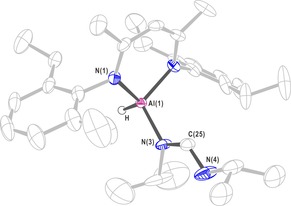
X‐ray single‐crystal structure of compound **6**. Anisotropic displacement parameters are depicted at the 50 % probability level. The hydrogen atoms are omitted for clarity, except for that of the Al−H bond. Selected bond lengths (Å) and angles (deg): Al(1)−N(1) 1.890(2), Al(1)−N(3) 1.844(3), N(3)−C(25) 1.363(5), N(4)−C(25) 1.275(5); C(25)‐N(3)‐Al(1) 120.3(3), N(4)‐C(25)‐N(3) 126.6(4).

To investigate the mechanism, the reaction of aluminum hydride complex **1** with one equivalent of DIC in toluene was carried out. It resulted in the expected aluminum formamidinate compound **6** by hydroalumination, which was characterized by single‐crystal X‐ray diffraction analysis (Figure 3). Meanwhile, a stoichiometric reaction of compound **1**, DIC, and HBpin was carried out. The appearance of the characteristic methine singlet (C−H) resonance at *δ*=7.68 ppm (N=C*H*) in the monohydroborated product could be identified in the ^1^H NMR spectrum.

According to Hill and co‐workers,^14^ a magnesium formamidinate ([CH{C(Me)NAr}_2_MgN(*i*Pr)CHN(*i*Pr)], containing a four‐membered ring was formed via a sigma Mg−N bond and a coordinate covalent Mg−N bond. This magnesium formamidinate compound was formed by the reaction between compound [HC{(Me)CN(2,6‐*i*Pr_2_C_6_H_3_)}_2_MgH]_2_ and DIC. However, aluminum formamidinate compound **6** was formed via a sigma Al−N bond.

**Scheme 4:**
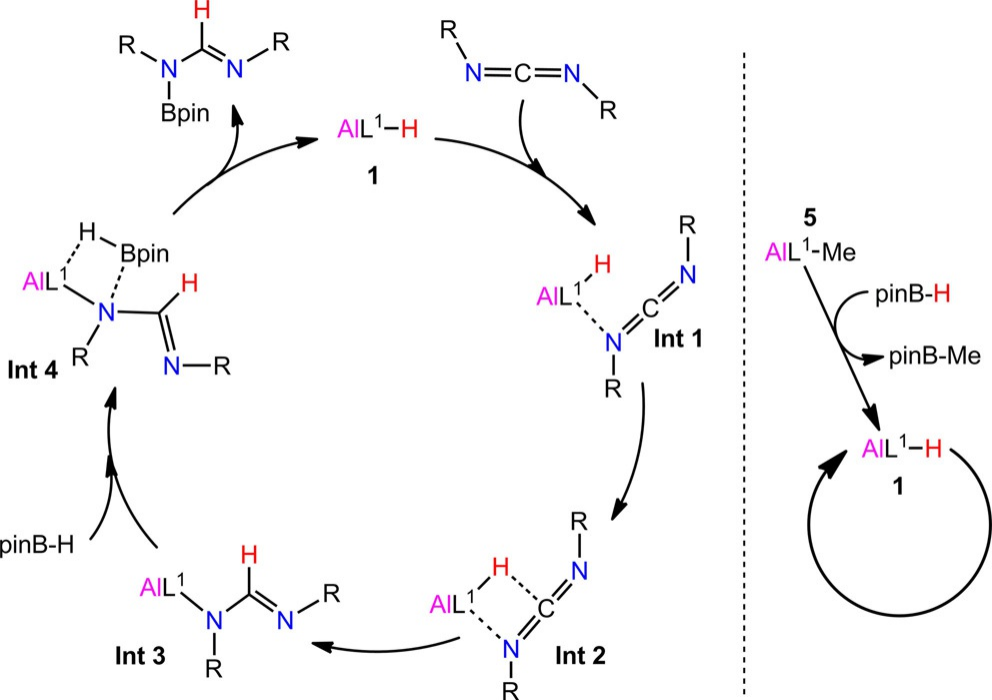
A suggested mechanism for the hydroboration of carbodiimides catalyzed by aluminum complexes (Left: aluminum hydride. Right: aluminum methyl).

On the basis of these experimental results and several mechanisms previously reported in the literature,3a, 8, 14, 16 the most possible mechanism for the hydroboration of carbodiimide with HBpin catalyzed by aluminum hydride complex is shown in Scheme 4 (left). Taking compound **1** as an example to describe the mechanism, compounds **2**–**4**, belonging to aluminum hydride compounds, have the same catalytic mechanism as that of compound **1**. Initially, a weakly bonded complex (**Int‐1**) was formed between active aluminum hydride compound **1** and carbodiimide. Subsequently, the carbodiimide substrate inserted into the Al−H bond with a hydride shift to the imide carbon atom through a four‐membered transition state (**Int‐2**). Then, an aluminum formamidinate compound (**Int‐3**) was formed via a sigma bond Al−N in essentially quantitative yield. In the final step, **Int‐3** reacted with a stoichiometric amount of pinacolborane (HBpin), during which, the Al−N bond in **Int‐3** and the B−H bond of pinacolborane followed a sigma bond exchange process to release the corresponding free *N*‐borylformamidine product with simultaneous regeneration of the active compound **1**.

The mechanism of the reaction catalyzed by aluminum alkyl complex **5** is shown in Scheme 4 (right). We presume that the Al−Me functions similar to compounds like Mg‐*n*Bu^14^ or Th‐Me^16^ in the hydroboration of carbodiimides. At first, aluminum pre‐catalyst **5** reacts with HBpin to form the active aluminum hydride compound **1**, which starts the catalytic cycle. The final catalytic process is similar to that described above. This process also suggests that **5** is less efficient and does not perform as well as **1**–**3** in the hydroboration of carbodiimides.

## Conclusions

This contribution describes the unprecedent aluminum‐catalyzed carbodiimide monohydroboration reactions and a suggested mechanism is explored and supported by an X‐ray diffraction analyzed aluminum formamidinatoborate **6** and an additional stoichiometric study.

The reduction reaction proceeds efficiently with HBpin and occurs only once under mild conditions with complete selectivity to give the monohydroborated *N*‐borylformamidine products. The electronic effect and steric hindrance of ligands on catalytic reactions are discussed. The monohydroborated C‐(B(OCMe_2_)_2_)‐2,6‐diisopropylphenylformamidinate can be formed with the extension of heating time, after reaching the maximum conversion. These results indicate the wide applicability of reactions catalyzed by aluminum compounds and reveal the possibility of reducing different substrates with different aluminum compounds.

## Experimental Section

Experimental details, ^1^H, ^13^C, ^11^B NMR spectra, crystal structure data and refinement details are given in Supporting Information. CCDC 1893454, 1893461, and 1893582 contain the supplementary crystallographic data for compounds **2**, **3**, and **6** in this paper. These data are provided free of charge by The Cambridge Crystallographic Data Centre.

## Conflict of interest

The authors declare no conflict of interest.

## Supporting information

As a service to our authors and readers, this journal provides supporting information supplied by the authors. Such materials are peer reviewed and may be re‐organized for online delivery, but are not copy‐edited or typeset. Technical support issues arising from supporting information (other than missing files) should be addressed to the authors.

Supplementary
