# Supplementary material for: Organoaluminum Compounds as Catalysts for Monohydroboration of Carbodiimides
Source: Chemistry. 2019 Aug 13;25(51):11918–23. doi: 10.1002/chem.201902000 (PMC6771511; doi:10.1002/chem.201902000)
Supplement: Supplementary file 1 — Supplementary [file CHEM-25-11918-s001.pdf]

CHEMISTRY
A European Journal

Supporting Information

Organoaluminum Compounds as Catalysts for Monohydroboration of Carbodiimides

Qiumiao Shen,[a] Xiaoli Ma,*[a] Wenling Li,[a] Wenqing Liu,[a] Yi Ding,[a] Zhi Yang,*[a] and Herbert W. Roesky*[b]

chem_201902000_sm_miscellaneous_information.pdf

Supporting information

Table of Contents

Experimental 3
General procedures 3
1. Synthesis of $L^2AlH_2(NMe_3)$ (2) 3
2. Synthesis of L^3AlH (3) 4
Hydroboration of carbodiimides 5
General procedure for hydroboration of carbodiimides 5
Figure S1. Calculation of the yield of hydroborated products. 5
1. Synthesis of intermediate compound 6 6
2. N-{$B(OCMe_2)_2$}-isopropylformamidinate (a) 6
3. N-{$B(OCMe_2)_2$}-cyclohexylformamidinate (b) 6
4. N-{$B(OCMe_2)_2$}-tert-butylformamidinate (c) 7
5. N-{$B(OCMe_2)_2$}-2,6-diisopropylphenylformamidinate (d) 7
6. C-{$B(OCMe_2)_2$}-2,6-diisopropylphenylformamidinate (e) 8
Stoichiometric reactions 9
Figure S2. Stoichiometric reactions of compound 1 with DIC 9
Figure S3. Stoichiometric reactions of compounds 3 and 5 with DIC 10
NMR spectra 11
Figure S4. ^1H NMR spectrum (400 MHz, 298 K, $CDCl_3$) of complex 2. 11
Figure S5. ^{13}C NMR spectrum (100 MHz, 298 K, $CDCl_3$) of complex 2. 11
Figure S6. ^1H NMR spectrum (400 MHz, 298 K, $CDCl_3$) of complex 3. 12
Figure S7. ^{13}C NMR spectrum (100 MHz, 298 K, $CDCl_3$) of complex 3. 12
Figure S8. ^1H NMR spectrum (400 MHz, 298 K, $CDCl_3$) of a. 13
Figure S9. ^{13}C NMR spectrum (100 MHz, 298 K, $CDCl_3$) of a. 13
Figure S10. ^{11}B NMR spectrum (128 MHz, 298 K, $CDCl_3$) of a. 14
Figure S11. ^1H NMR spectrum (400 MHz, 298 K, $CDCl_3$) of b. 14
Figure S12. ^{13}C NMR spectrum (100 MHz, 298 K, $CDCl_3$) of b. 15
Figure S13. ^{11}B NMR spectrum (128 MHz, 298 K, $CDCl_3$) of b. 15
Figure S14. ^1H NMR spectrum (400 MHz, 298 K, $CDCl_3$) of c. 16
Figure S15. ^{13}C NMR spectrum (100 MHz, 298 K, $CDCl_3$) of c. 16
Figure S16. ^{11}B NMR spectrum (128 MHz, 298 K, $CDCl_3$) of c. 17
Figure S17. ^1H NMR spectrum (400 MHz, 298 K, $CDCl_3$) of d. 17
Figure S18. ^{13}C NMR spectrum (100 MHz, 298 K, $CDCl_3$) of d. 18
Figure S19. ^{11}B NMR spectrum (128 MHz, 298 K, $CDCl_3$) of d. 18
Figure S20. ^1H NMR spectrum (400 MHz, 298 K, $CDCl_3$) of e. 19
Figure S21. ^{13}C NMR spectrum (100 MHz, 298 K, $CDCl_3$) of e. 19
Figure S22. ^{11}B NMR spectrum (128 MHz, 298 K, $CDCl_3$) of e. 20

Figure S23.^{1}H NMR spectrum (400 MHz, 298 K, $CDCl_3$) of complex 6. ..20
Figure S24.^{13}C NMR spectrum (100 MHz, 298 K, $CDCl_3$) of complex 6. .21
Table S1: Crystal structure data and refinement details for complexes 2, 3 and 6. 22
References...23

Experimental

General procedures

All manipulations were carried out under a purified nitrogen atmosphere using Schlenk techniques or inside an Etelux MB 200G glovebox. All solvents were refluxed with the appropriate drying agent and distilled prior to use. Commercially available chemicals were purchased from J&K chemical or VAS and used as received. Compounds **1**,[1] **4**,[2] and **5**[3] were prepared as previously described in the literature. $\mathbf{L^2}$ was obtained from the reaction of 2, 6-diethylbenzenamine and *o*-fluorobenzaldehyde according to previously reported procedures.[2, 4] Elemental analyses were performed by the Analytical Instrumentation Center of the Beijing Institute of Technology. NMR spectra were recorded on Bruker AM 400 spectrometer. Melting points were measured in sealed glass tubes.

Synthesis of $\mathbf{L^3}$ 2, 6-bis(1-methylethyl)-N-(2-pyridinyl methylene)phenyl amine: [5, 6] A solution of 2-pyridinecarboxaldehyde (20 mmol), 2, 6-diisopropylaniline (20 mmol), and a catalytic amount of formic acid in methanol (15 mL) was stirred at room temperature overnight. The solvent was evaporated on a rotary evaporator to remove volatile components, crude product was washed with water to remove residual formic acid, the product was recrystallized from *n*-hexane to yellow crystals, which were filtered off and washed with cold *n*-hexane.

1. Synthesis of $L^2AlH_2(NMe_3)$ (2) [2]

$H_3Al{\cdot}NMe_3$ (1 M in toluene, 1 mL, 1 mmol) was added drop by drop into a toluene solution (20 mL) of $\mathbf{L^2}$ (0.255 g, 1 mmol) at 0 °C. After the addition was complete, the reaction mixture was allowed to warm to room temperature and stirring was continued for 24 h. The solution was evaporated to dryness in vacuo, and the residue was dissolved in *n*-hexane. Then the solution was filtered. The filtrate was concentrated and stored at -20 °C in a freezer for 3 days to afford compound **2** as colorless crystals. An additional crop of **2** was obtained from the mother liquor. Total yield 0.302 g (88%); m.p. 63-66 °C. ^{1}H NMR (400 MHz, $CDCl_3$, 298 K, TMS) δ_H (ppm): 7.07-6.75 (m, 7 H, Ar-H), 4.22 (s, 2 H, CH_2N), 2.60-2.45 (m, 4 H, CH_2Me), 2.39 (s, 9 H, NMe_3), 0.98 (t, J = 7.6 Hz, 6 H, CH_3); ^{13}C NMR (100 MHz, $CDCl_3$, 298 K, TMS) δ_C (ppm): 146.84, 141.36, 131.01 , 127.99, 126.97, 124.54, 122.25, 122.22, 122.19, 113.96, 113.73 (C of Ar), 49.60 (CH_2), 46.07, 22.87, 14.05($CH(CH_3)_2$). Anal. Calcd for $C_{20}H_{30}AlFN_2$ (344.44): C, 69.74; H, 8.78; N, 8.13. Found: C, 69.47; H, 8.93; N, 8.41.

2. Synthesis of L^3AlH (3)

$H_3Al·NMe_3$ (1 M in toluene, 1 mL, 1 mmol) was added drop by drop into a toluene solution (20 mL) of $\mathbf{L^3}$ (0.266 g, 1.0 mmol) at 0 °C. After the addition was complete, the reaction mixture was allowed to warm to room temperature and stirring was continued for 24 h. Then the solution was filtered. The filter residue was washed with cold n-hexane. The filtrate was concentrated and stored at –20 °C in a freezer for 1 day to afford compound **3** as yellow crystals. An additional crop of 3 was obtained from filter residue and mother liquor. Total yield 0.40 g (72%); m.p. 160-162 °C. ^{1}H NMR (400 MHz, $CDCl_3$, 298 K, TMS) δ_H (ppm): 7.48 (m, 2 H, Py-H), 7.42 (d, J = 5.5 Hz, 2 H, Py-H), 7.11 (d, J = 7.7 Hz, 4 H, Ph-H), 6.94 (t, J = 7.6 Hz, 2 H, Ph-H), 6.68 (dd, J = 15.6, 7.9 Hz, 4 H, Py-H), 4.64(d, J = 19.6 Hz, 2 H, NCH_2), 4.33(d, J = 19.6 Hz, 2 H, NCH_2), 3.67(tt, J = 13.7, 6.9 Hz, 4 H, $CHMe_2$), 1.34(d, J = 6.8 Hz, 6 H, $(CH_3)_2$), 1.16(d, J = 6.9 Hz, 6 H, $(CH_3)_2$), 1.05(d, J = 6.8 Hz, 6 H, $(CH_3)_2$), 0.28(d, J = 7.0 Hz, 6 H, $(CH_3)_2$); ^{13}C NMR (100 MHz, $CDCl_3$, 298 K, TMS) δ_C (ppm): 160.43, 148.96, 147.57, 147.33, 144.19, 136.12 , 122.88 , 122.49, 121.46, 120.09, 119.78 (C of Ar and Py), 57.73 (CH_2), 26.24 , 25.15, 23.83, 21.32($CH(CH_3)_2$). Anal. Calcd for $C_{36}H_{47}AlN_4$ (562.78): C, 76.83; H, 8.42; N, 9.96. Found: C, 77.13; H, 8.53; N, 9.80.

Hydroboration of carbodiimides

General procedure for hydroboration of carbodiimides

HBpin (0.14 g, 1.1 mmol) and carbodiimide (1 mmol) were loaded in a dried sealable J-Young Tube under nitrogen atmosphere inside the glove box, followed by the addition of the desired catalyst (0.04 mmol). Samples were taken out of the glove box and stirred and heated to a certain temperature in an oil bath. The reaction time depends on the nature of the starting materials. The reaction was terminated by exposing the mixture to air. The products were analyzed by using ^{1}H NMR, ^{13}C NMR, ^{11}B NMR.

The yield of hydroborated products was calculated from the ratio of starting material and the target product by ^{1}H NMR and using the internal standard PhOMe shows the same results. For example, the hydroboration of DippNCNDipp catalyzed by compound **1** at 80℃ was conducted, after 1 h to the reaction mixture was added equimolar amount of PhOMe, and dissolved in $CDCl_3$. Figure S1 (bottom) shows the reaction result, in which the integral value of the B peak (CH_3 of PhOMe) is set to 3, so the integral value of the A (NC*H*N of **d**) is the yield of hydroborated products. So we also can easily calculate the yield of **d** from the ratio of C ($CH(CH_3)_2$ of DippNCNDipp) and D ($CH(CH_3)_2$ of **d**) peaks. The same reaction for 2h was conducted. Figure S1 (top) shows the result, C peak disappeared compared to the bottom one. (A: NC*H*N of **d** ; B: CH_3 of PhOMe ; C: $CH(CH_3)_2$ of DippNCNDipp ; D: $CH(CH_3)_2$ of **d**).

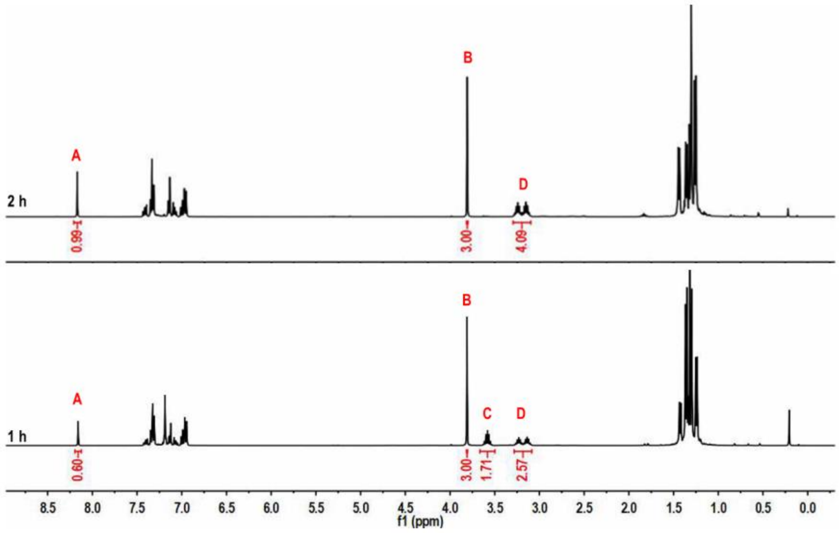

Figure S1. Calculation of the yield of hydroborated products.

1. Synthesis of intermediate compound 6

Compound **1** (1 mmol, 0.392 g) and N, N'-diisopropylcarbodiimide (1 mmol, 0.126 g) was added in a dried Schlenk flask inside the glove box, then dissolved in 20 ml toluene. The solution was stirred and heated at 80 ℃ for 10 h, then concentrated and stored at 0-4 ℃ for 3 days to afford compound **6** as colorless crystals. Total yield 0.41 g (80%). m.p. 163-166 °C. ^{1}H NMR (400 MHz, $CDCl_3$, 298 K, TMS) δ_H (ppm): 7.46 (s, 1 H), 7.08 (m, 6 H, Ar-H), 5.16 (s, 1 H, CH), 3.09 (m, 1 H, $CH(CH_3)_2$), 2.75-2.42 (m, 8 H,CH_2), 2.41-2.30 (m, 1 H, $CH(CH_3)_2$), 1.75-1.60 (s, 6 H, CH_3), 1.14 (t, J = 7.6 Hz, 12 H, CH_3), 1.05-0.60 (m, 12 H, CH_3) ppm. ^{13}C NMR (100 MHz, $CDCl_3$, 298 K, TMS) δ_C (ppm): 169.19, 168.46, 154.52, 139.77, 137.44, 125.54, 124.90, 124.59, 95.87, 56.84, 50.83, 42.38, 24.87, 23.22, 22.85, 22.78, 22.66, 22.15, 21.87, 12.81, 12.50 ppm. Anal. Calcd for $C_{32}H_{49}AlN_4$ (516.74): C, 74.38; H, 9.56; N, 10.84. Found: C, 74.60; H, 9.44; N, 10.69.

2. N-{B(OCMe$_2$)$_2$}-isopropylformamidinate (a) [7]

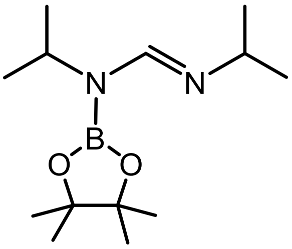

The hydroboration reaction between N, N'-diisopropylcarbodiimide (1 mmol, 0.126 g) and pinacolborane (1.1 mmol, 0.14 g) was carried out according to the general procedure. The yields of the product are given in Table 1, 2 of the manuscript. The same yields were observed using 1 mmol of PhOMe. ^{1}H NMR (400 MHz, $CDCl_3$, 298 K, TMS) δ_H (ppm): 7.74 (s, 1H, NCH), 4.38 (dt, J = 13.3, 6.6 Hz, 1H, $BNCH(CH_3)_2$), 3.24 (dt, J = 12.3, 6.1 Hz, 1H, $NCH(CH_3)_2$), 1.17 (s, 12H, $C(CH_3)_2$) 1.14(d, J = 6.9 Hz, 6H, $BNCH(CH_3)_2$), 1.03 (d, J = 6.3 Hz, 6H, $NCH(CH_3)_2$). ^{13}C NMR (100 MHz, $CDCl_3$, 298 K, TMS) δ_C (ppm): 149.34 (NCHN), 81.59 ($OC(CH_3)_2$), 55.84 ($BNCH(CH_3)_2$), 41.99 ($NCH(CH_3)_2$), 24.19 ($BNCH(CH_3)_2$), 23.48($C(CH_3)_2$), 20.44 ($CH(CH_3)_2$). ^{11}B NMR (128 MHz, $CDCl_3$, 298 K, TMS) δ_B (ppm): 25.03 (s, NB).

3. N-{B(OCMe$_2$)$_2$}-cyclohexylformamidinate (b) [7]

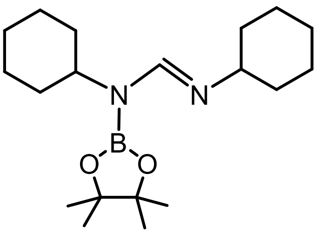

The hydroboration reaction between N,N'-dicyclohexylcarbodiimide (1 mmol, 0.226 g) and pinacolborane (1.1 mmol, 0.14 g) was carried out according to the

general procedure. The yields of the product are given in Table 1, 2 of the manuscript. The same yields were observed using 1 mmol of PhOMe. ^{1}H NMR (400 MHz, $CDCl_3$, 298 K, TMS) δ_H (ppm): 7.77 (s, 1H, NCH), 3.97 (m, 1H, $BNCH(CH_2)_2$), 2.86 (m,1H, $NCH(CH_2)_2$), 1.68 (dd, J = 18.3, 15.3 Hz, 6H, Cy-H), 1.52 (t, J = 10.3 Hz, 6H, Cy-H), 1.34-1.19 (m, 6H, Cy-H),1.15 (s, 12H, $C(CH_3)_2$), 1.12-1.01 (m, 4H, Cy-H). ^{13}C NMR (100 MHz, $CDCl_3$, 298 K, TMS) δ_C (ppm): 149.53 (NCHN), 81.48 ($C(CH_3)_2$), 64.01 ($BNCH(CH_2)_2$), 49.96 ($NCH(CH_2)_2$), 34.48 (BNCH(CH2)2(CH2)2CH2), 30.35 ($NCH(CH_2)_2(CH_2)_2CH_2$), 25.29 ($BNCH(CH_2)_2(CH_2)_2CH_2$), 24.81 ($BNCH(CH_2)_2$ $(CH_2)_2CH_2$), 24.72 ($NCH(CH_2)_2(CH_2)_2CH_2$), 24.14 ($NCH(CH_2)_2(CH_2)_2CH_2$), 23.28 ($C(CH_3)_2$). ^{11}B NMR (128 MHz, $CDCl_3$, 298 K, TMS) δ_B (ppm): 24.77 (s, NB).

4. N-{B(OCMe$_2$)$_2$}-tert-butylformamidinate (c) [7]

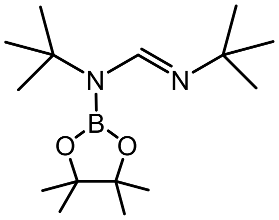

The hydroboration reaction between N,N'-di-tert-butylcarbodiimide (1 mmol, 0.154 g) and pinacolborane (1.1 mmol, 0.14 g) was carried out according to the general procedure. The yields of the product are given in Table 1, 2 of the manuscript. The same yields were observed using 1 mmol of PhOMe. ^{1}H NMR (400 MHz, $CDCl_3$, 298 K, TMS) δ_H (ppm): 7.757 (1H, s, NCH), 1.22 (18H, s, $C(CH_3)_3$), 1.137 (12H, s, $C(CH_3)_2$). ^{13}C NMR (100 MHz, $CDCl_3$, 298 K, TMS) δ_C (ppm): 147.49 (NCHN), 80.87 ($C(CH_3)_2$), 53.45 ($C(CH_3)_3$), 29.18 ($C(CH_3)_3$), 23.43($C(CH_3)_2$). ^{11}B NMR (128 MHz, $CDCl_3$, 298 K, TMS) δ_B (ppm): 25.285 (s, NB).

5. N-{B(OCMe$_2$)$_2$}-2,6-diisopropylphenylformamidinate (d) [7]

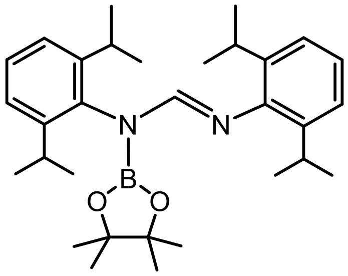

The hydroboration reaction between N, N'-2,6-diisopropylphenylcarbodiimide (1 mmol, 0.362 g) and pinacolborane (1.1 mmol, 0.14 g) was carried out according to the general procedure. The yields of the product are given in Table 1 of the manuscript. The same yields were observed using 1 mmol of PhOMe. ^{1}H NMR (400 MHz, $CDCl_3$, 298 K, TMS) δ_H (ppm): 8.05 (s, 1H, NCHN), 7.35 – 7.29 (m, 1H Ar-H), 7.23 (d, J = 7.6 Hz, Ar -H), 7.03 (d, J = 6.8 Hz, 2H, Ar -H), 7.00 – 6.94 (m, 1H, Ar -H), 3.12 (dt, J = 13.6, 6.8 Hz, 2H, $CH(CH_3)_2$), 3.03 (dt, J = 13.7, 6.9 Hz, 2H, $CH(CH_3)_2$), 1.32 (d, J = 6.9 Hz, 6H, $CH(CH_3)_2$), 1.24 (d, J = 6.8 Hz, 6H, $CH(CH_3)_2$), 1.21 (s, 12H, $C(CH_3)_2$), 1.14 (d, J = 7.0 Hz, 12H, $CH(CH_3)_2$). ^{13}C NMR (100 MHz, $CDCl_3$, 298 K, TMS) δ_C (ppm): 151.88 (Ar -C), 146.86 (Ar -C), 144.64 (Ar -C), 138.54 (Ar -C), 132.17 (Ar

-C), 127.15 (Ar -C), 122.53 (Ar -C), 122.10 (Ar -C), 121.52(Ar -C) 82.92 ($C(CH_3)_2$), 27.51 ($CH(CH_3)_2$), 26.28 (($CH(CH_3)_2$), 24.02 ($CH(CH_3)_2$), 23.39 ($CH(CH_3)_2$), 22.89 ($CH(CH_3)_2$), 22.07($CH(CH_3)_2$). ^{11}B NMR (128 MHz, $CDCl_3$, 298 K, TMS) δ_B (ppm): 24.94 (s, NB).

6. C-{B($OCMe_2$)$_2$}-2,6-diisopropylphenylformamidinate (e)

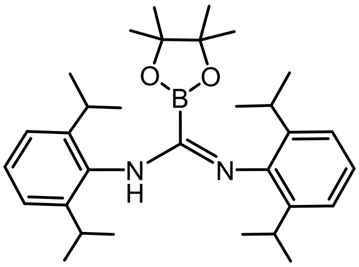

N, N'-2,6-diisopropylphenylcarbodiimide (1 mmol, 0.362 g), HBpin (2 mmol, 0.25 g) and compound 4 (0.04 mmol, 0.021 g) were loaded in a dried J-Young Tube under nitrogen atmosphere inside the glove box. The tube was sealed, kept in an oil bath at 80℃ for 4 days. After the reaction is complete, the mixture was exposed to air and added to n-hexane (5 mL). Filtration of the mixture and washing the white filter residue with cold n-hexane (5-10 mL) to obtain the final product. ^{1}H NMR (400 MHz, $CDCl_3$, 298 K, TMS) δ_H (ppm): 9.72 (s, 1H, NCHN), 7.34 (dd, J = 15.8, 8.1 Hz, 2H, Ar-H), 7.21 (d, J = 7.7 Hz, 2H, Ar-H), 7.17 (d, J = 7.7 Hz, 2H, 2H, Ar -H), 3.20 (hept, J = 6.8 Hz, 2H, $CH(CH_3)_2$), 3.06-2.94 (m, 2H, $CH(CH_3)_2$), 1.29 (s, 12H, $C(CH_3)_2$), 1.28-1.18 (m, 24H, $CH(CH_3)_2$). ^{13}C NMR (100 MHz, $CDCl_3$, 298 K, TMS) δ_C (ppm): 187.41 (B-C), 145.21 (Ar-C), 144.91 (Ar-C), 136.15 (Ar-C), 133.61 (Ar-C), 127.27 (Ar-C), 126.93 (Ar-C), 122.63 (Ar-C), 122.28 (Ar-C),84.01 ($C(CH_3)_2$), 27.89 ($CH(CH_3)_2$), 27.72 (($CH(CH_3)_2$), 23.37 ($CH(CH_3)_2$), 23.30 ($CH(CH_3)_2$), 23.14 ($CH(CH_3)_2$), 22.01 ($CH(CH_3)_2$). ^{11}B NMR (128 MHz, $CDCl_3$, 298 K, TMS) δ_B (ppm): 23.92 (s, NB).

Stoichiometric reactions

In a J. Young tube, 0.5 mmol aluminum complex **1** (0.1965 g) and 1.0 equivalent DIC (0.063 g) was diluted with 0.5 mL $CDCl_3$. The reaction mixture was stirred at 60 °C for 12h. And appearance of a characteristic singlet of aluminum formamidinate compound **6** at δ= 7.458 ppm was observed after 3h. 1.0 equivalent HBpin was added after 5h. After heating for 6 hours, the resonance appeared at δ = 7.68, which was compared to product **a**, assigned to the N=C*H*N unit of the expected final formamidinate product. With the extension of heating time, **6** gradually decomposed to produce the final product. The splitting of the resonance signal of compound **1** at δ = 5.33 and 1.64 ppm also indicates the occurrence of hydroalumination and the generation of aluminum formamidinate compound.

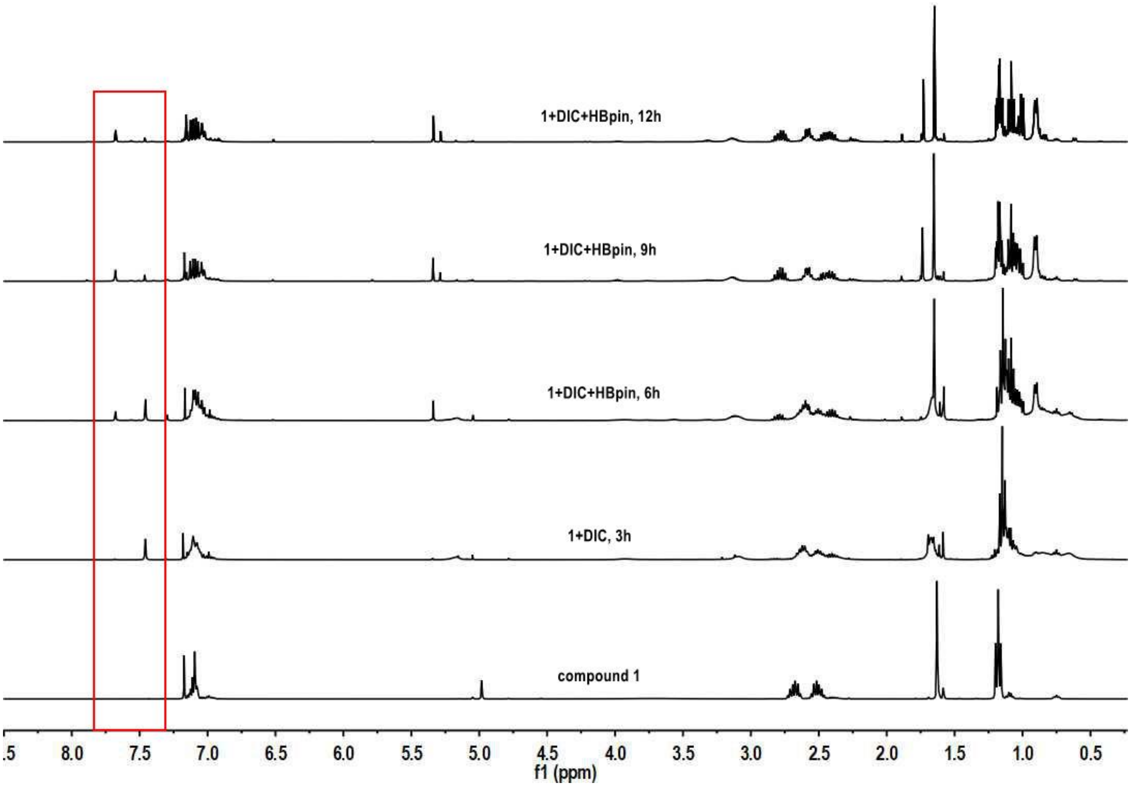

Figure S2. Stoichiometric reactions of compound 1 with DIC

In a J. Young tube, 0.5 mmol aluminum complex **3** (0.281 g) and 1.0 equivalent DIC (0.063 g) was diluted with 5 ml Tol. The reaction mixture was stirred at 60 °C for 6h. And a characteristic singlet at δ= 8.560 ppm was observed. We suppose that it might be N=CHN unit, which may indicate that compound **3** reacts with DIC to produce an aluminum formamidinate via hydroalumination. Then in a J. Young tube, 0.5 mmol aluminum complex **5** (0.209 g) and 1.0 equivalent DIC (0.063 g) was

diluted with 5 ml Tol. The reaction mixture was stirred at 60 °C for 6 h. The splitting of the resonance signal of compound **5** at δ = 1.151 and 1.636 ppm might indicate the formation of compound **5** with DIC.

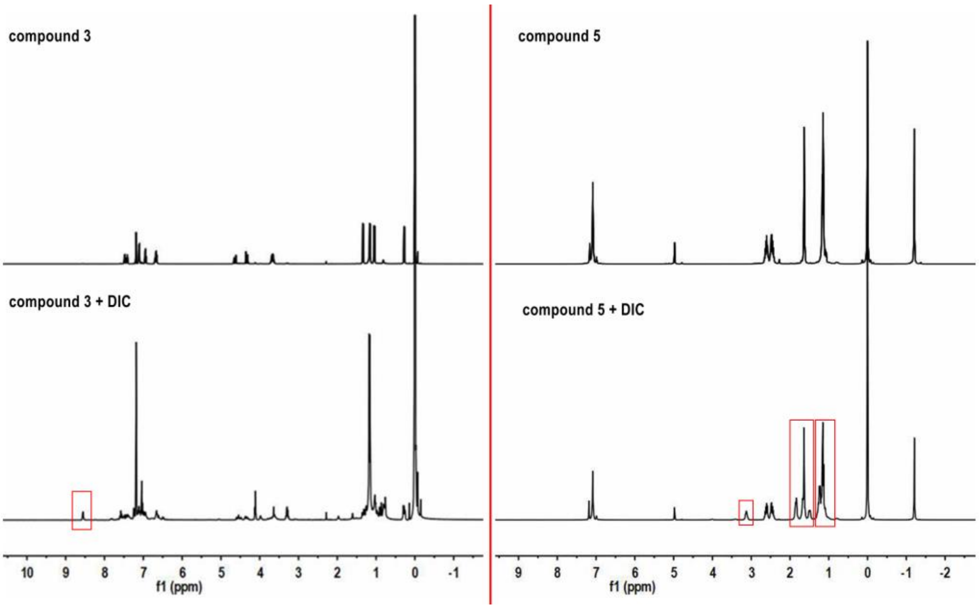

Figure S3. Stoichiometric reactions of compounds 3 and 5 with DIC

NMR spectra

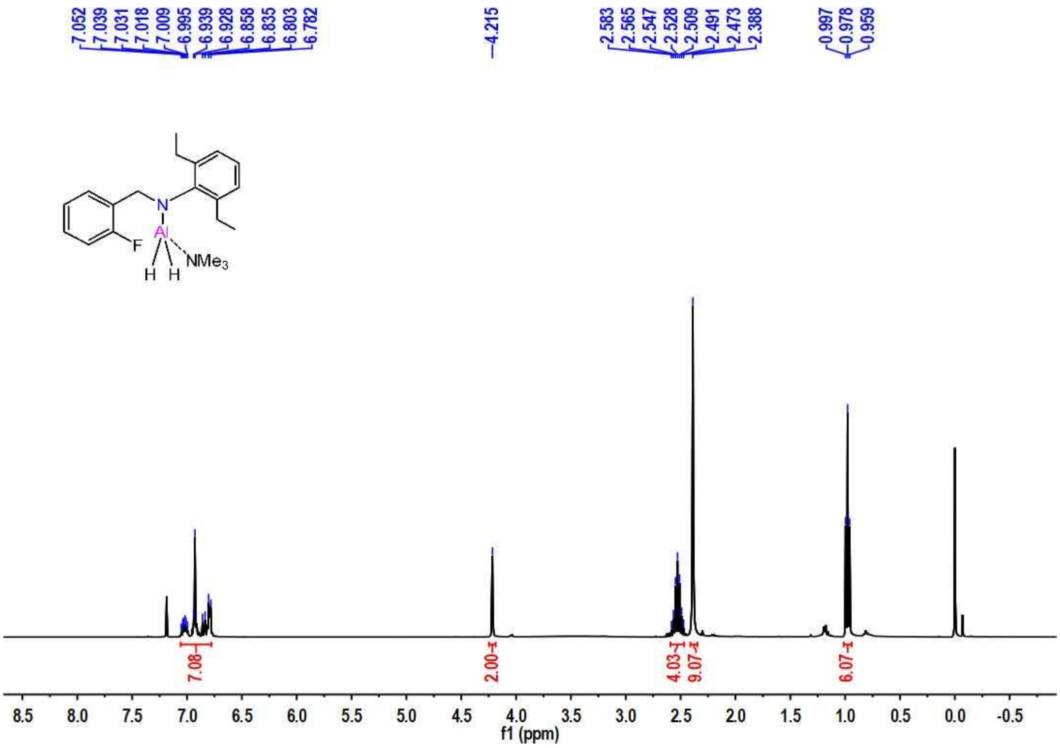

Figure S4.[1]**H NMR spectrum (400 MHz, 298 K, CDCl$_3$) of complex 2.**

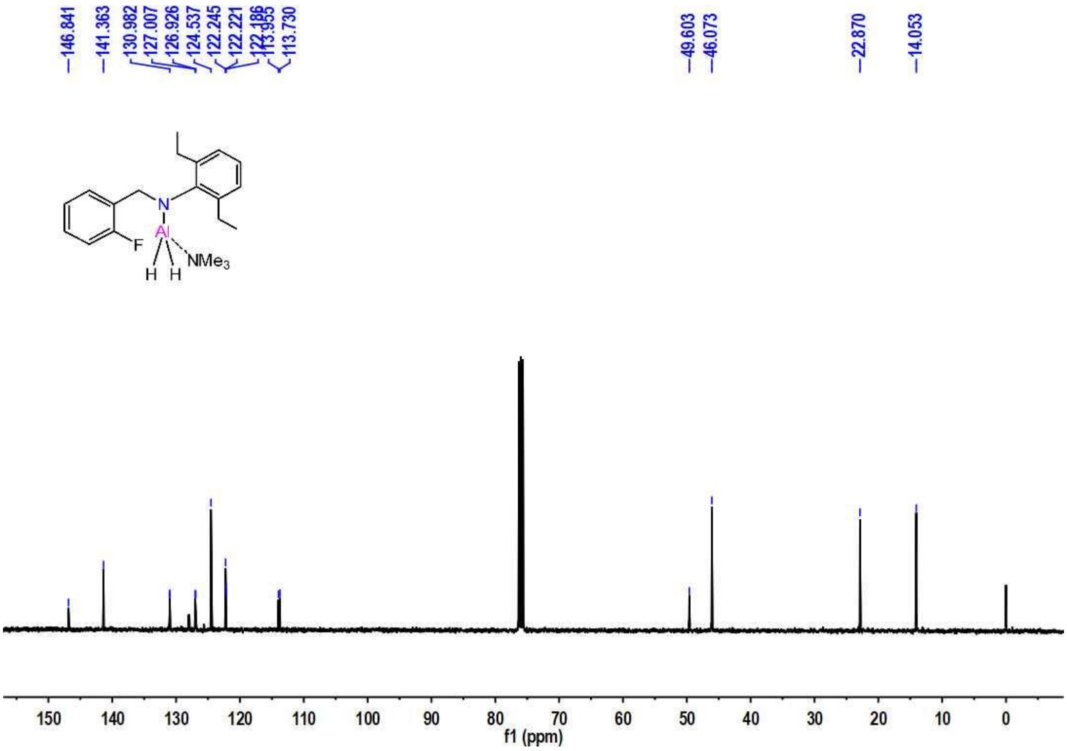

Figure S5.[13]**C NMR spectrum (100 MHz, 298 K, CDCl$_3$) of complex 2.**

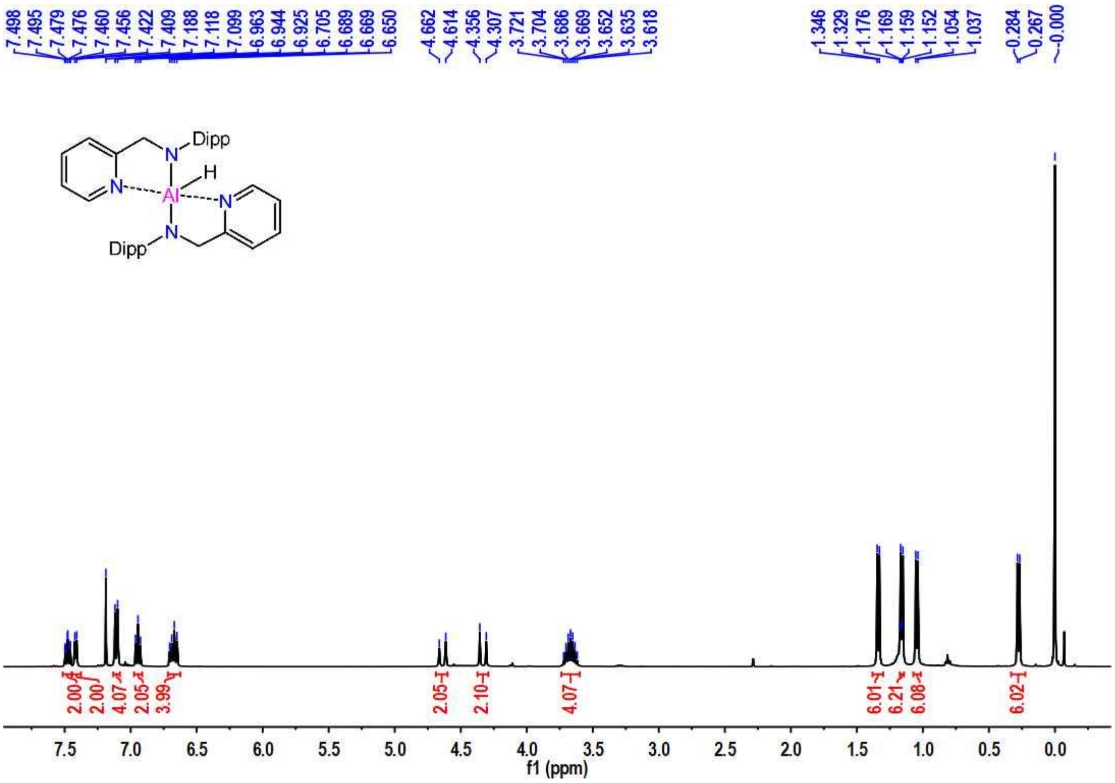

Figure S6.^{1}H NMR spectrum (400 MHz, 298 K, $CDCl_3$) of complex 3.

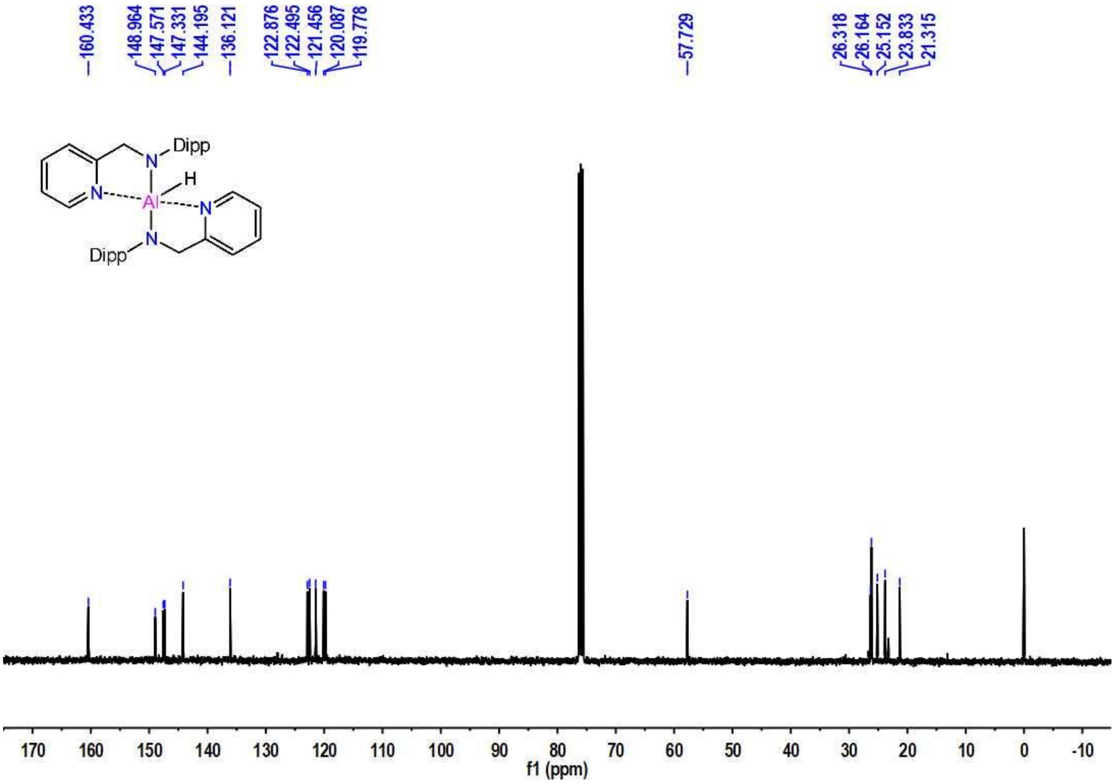

Figure S7.^{13}C NMR spectrum (100 MHz, 298 K, $CDCl_3$) of complex 3.

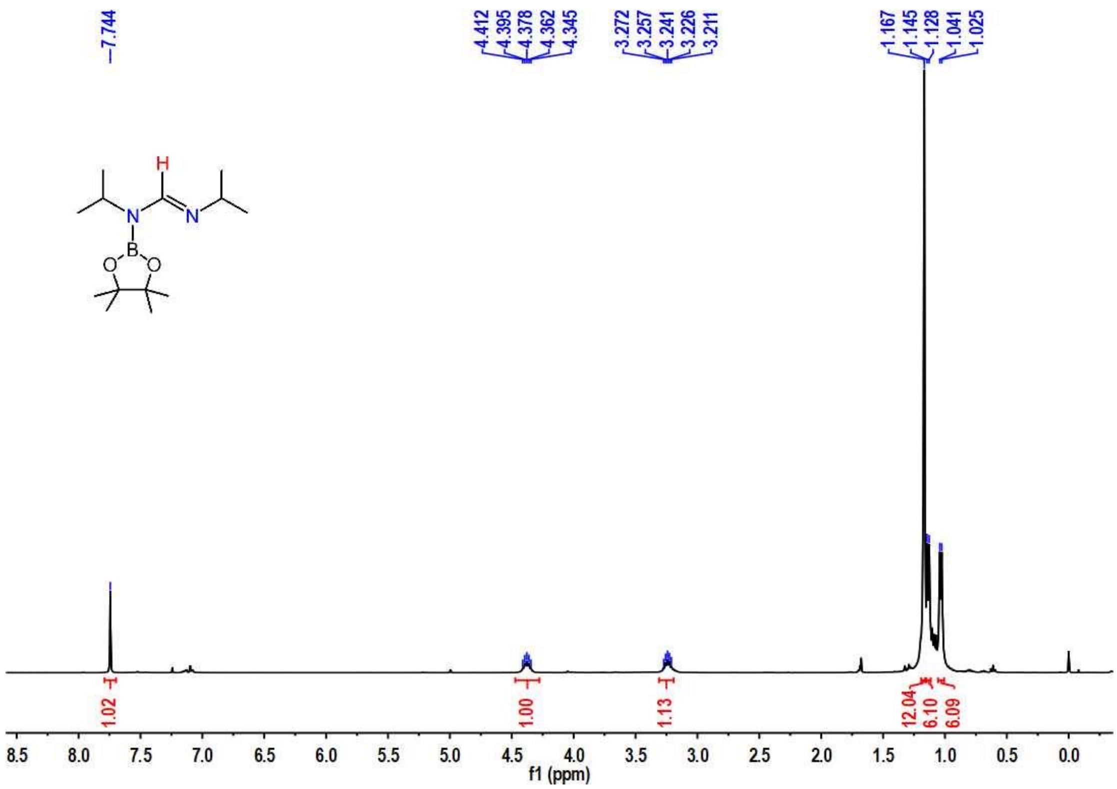

Figure S8.^{1}H NMR spectrum (400 MHz, 298 K, CDCl3) of a.

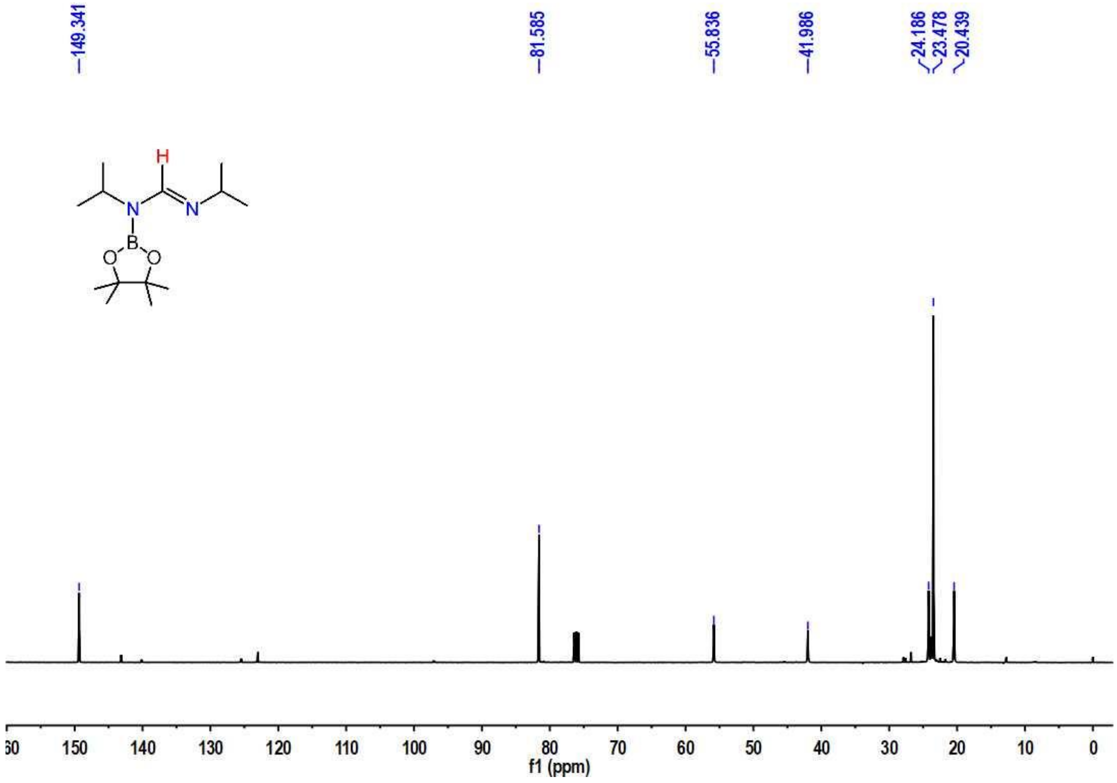

Figure S9. ^{13}C NMR spectrum (100 MHz, 298 K, CDCl3) of a.

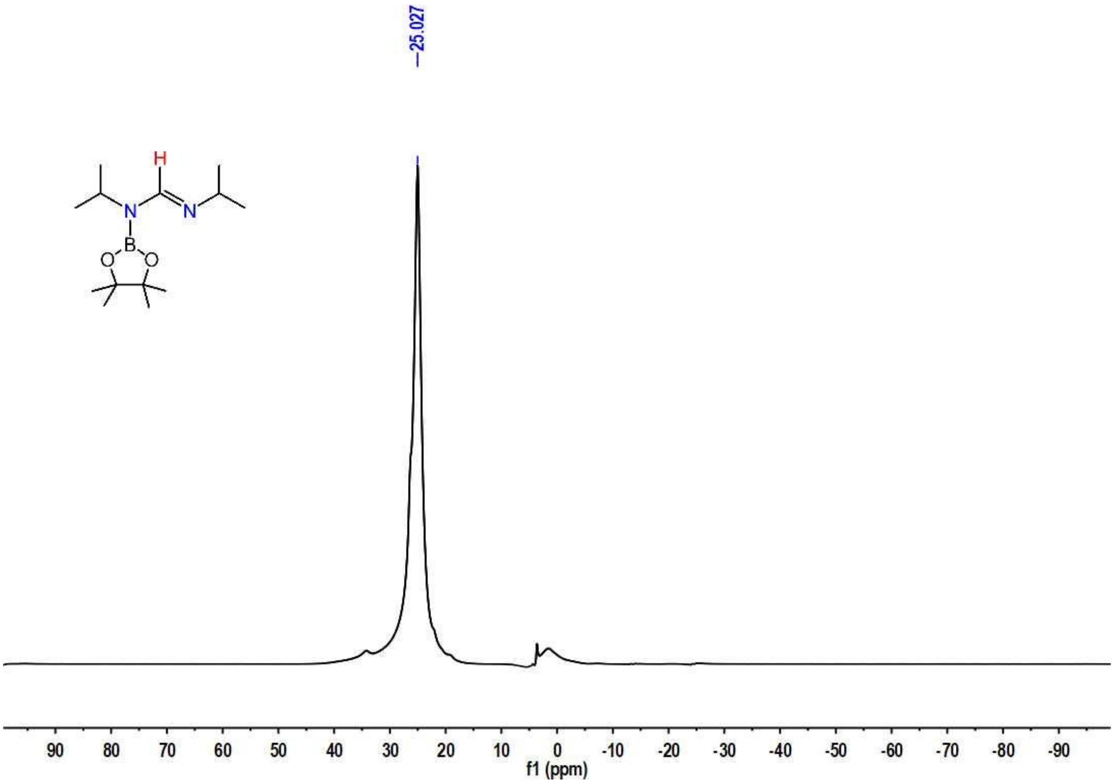

Figure S10. ^{11}B NMR spectrum (128 MHz, 298 K, $CDCl_3$) of a.

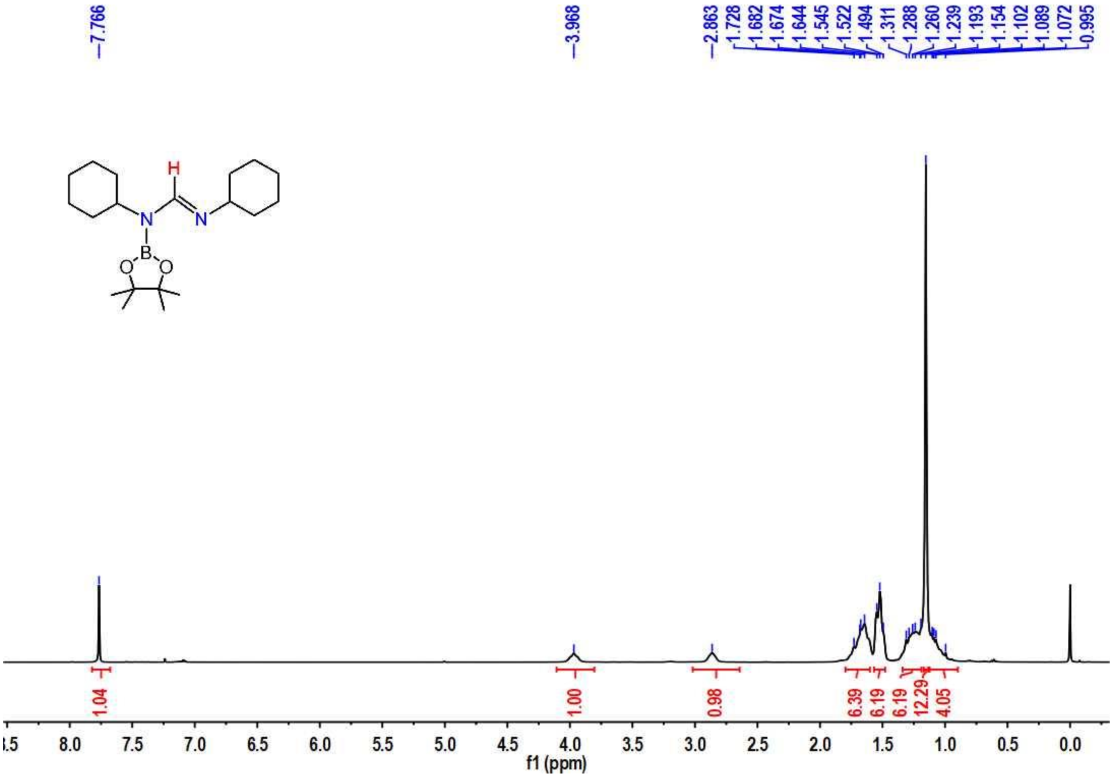

Figure S11.^{1}H NMR spectrum (400 MHz, 298 K, $CDCl_3$) of b.

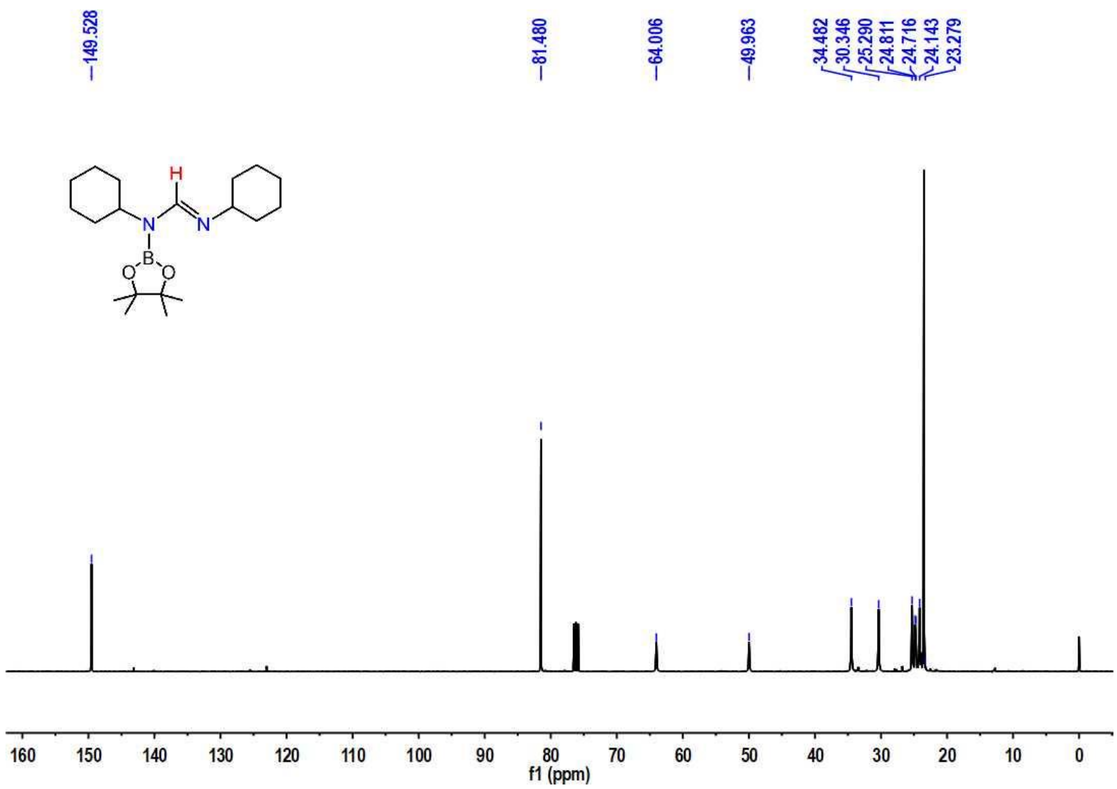

Figure S12. ^{13}**C NMR spectrum (100 MHz, 298 K, $CDCl_3$) of b.**

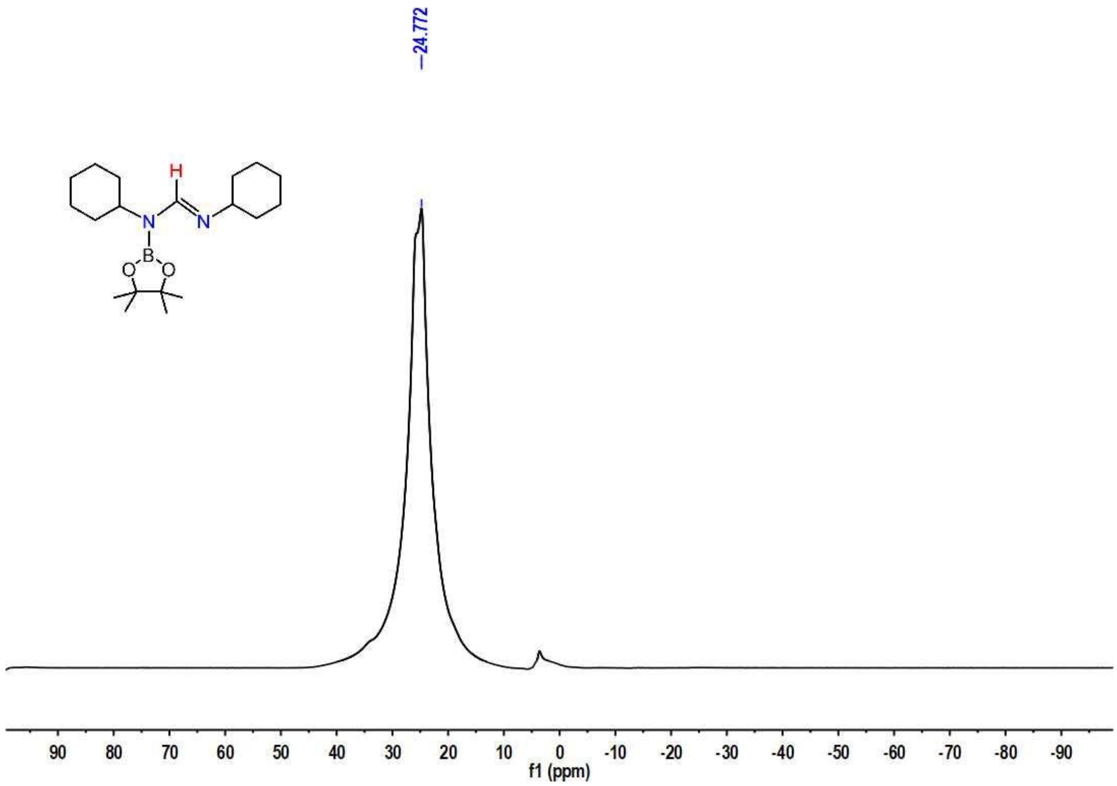

Figure S13. ^{11}**B NMR spectrum (128 MHz, 298 K, $CDCl_3$) of b.**

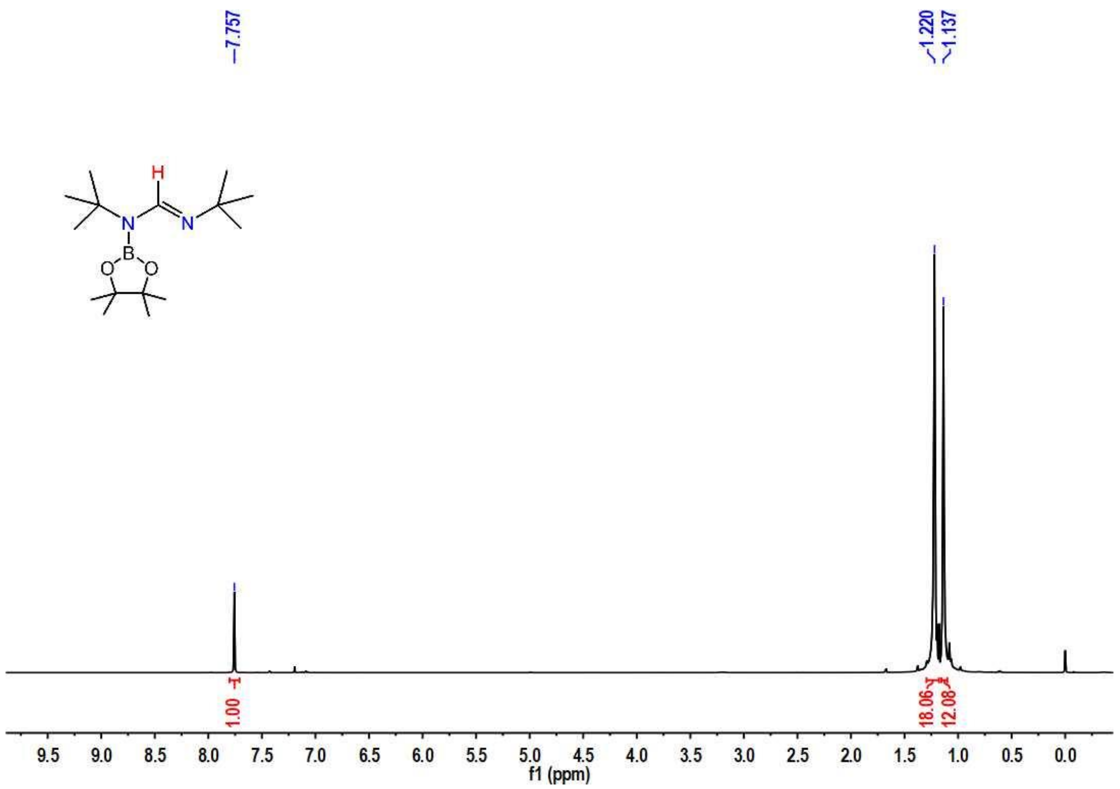

Figure S14.[1]**H NMR spectrum (400 MHz, 298 K, $CDCl_3$) of c.**

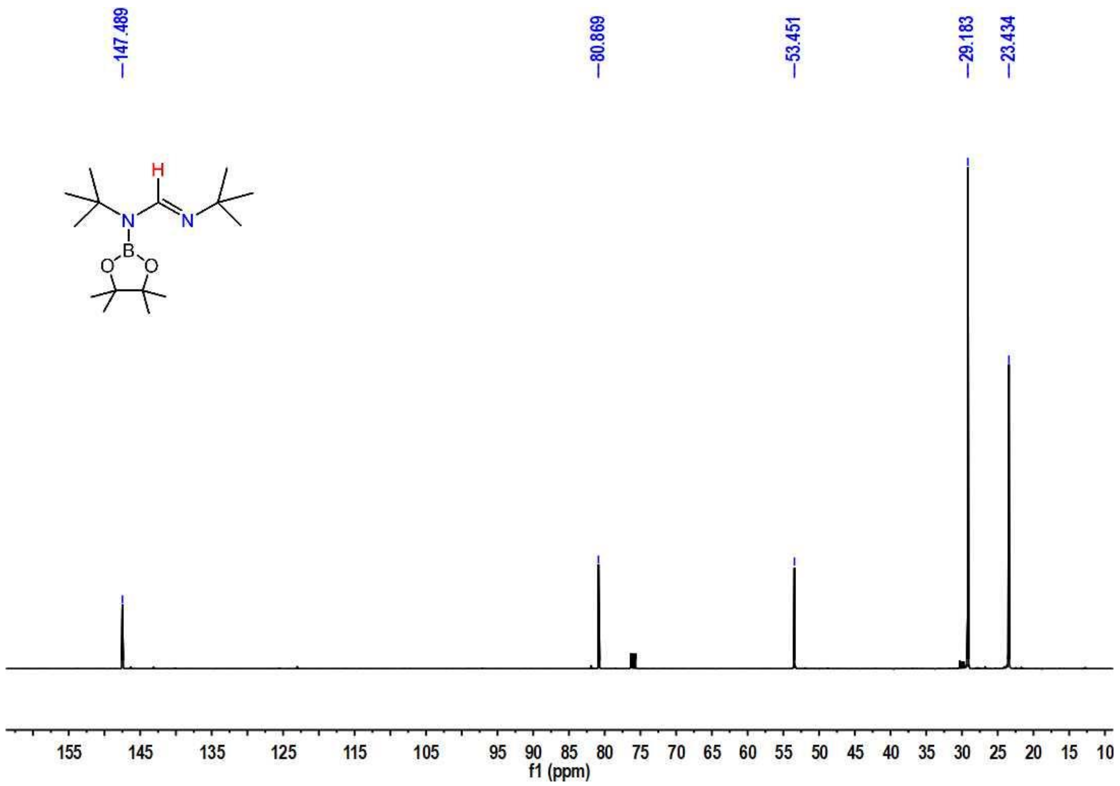

Figure S15. ^{13}**C NMR spectrum (100 MHz, 298 K, $CDCl_3$) of c.**

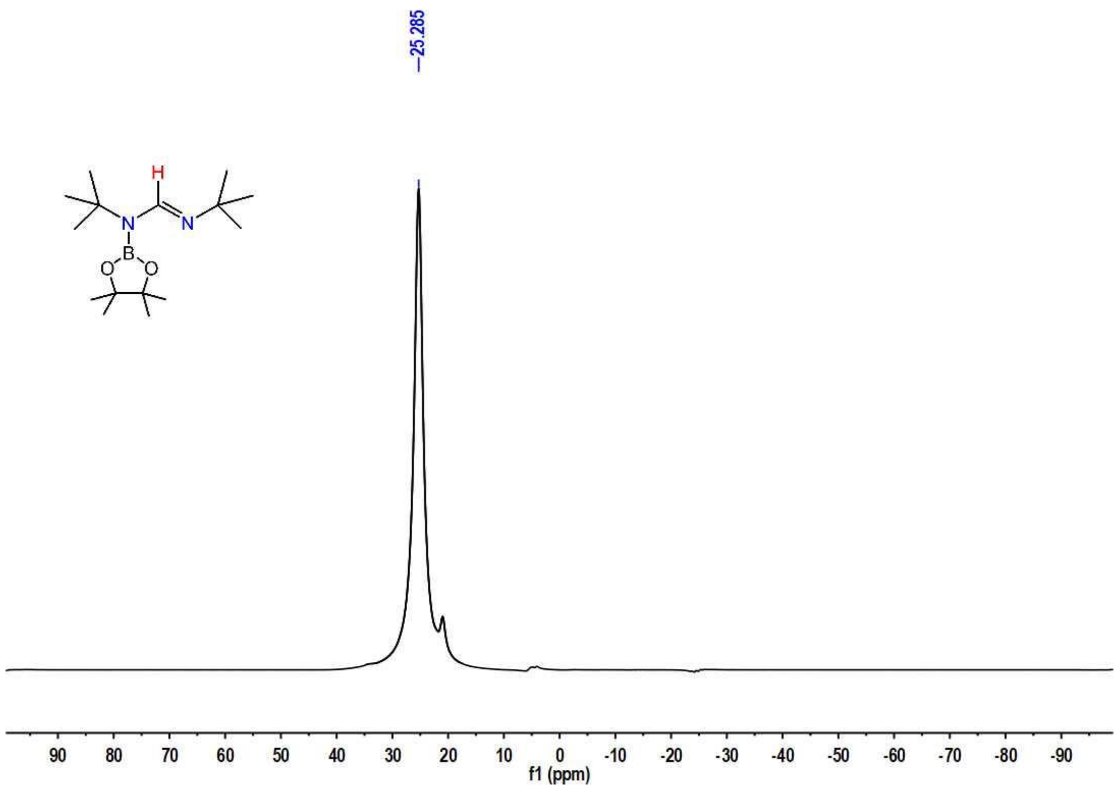

Figure S16. ^{11}B NMR spectrum (128 MHz, 298 K, $CDCl_3$) of c.

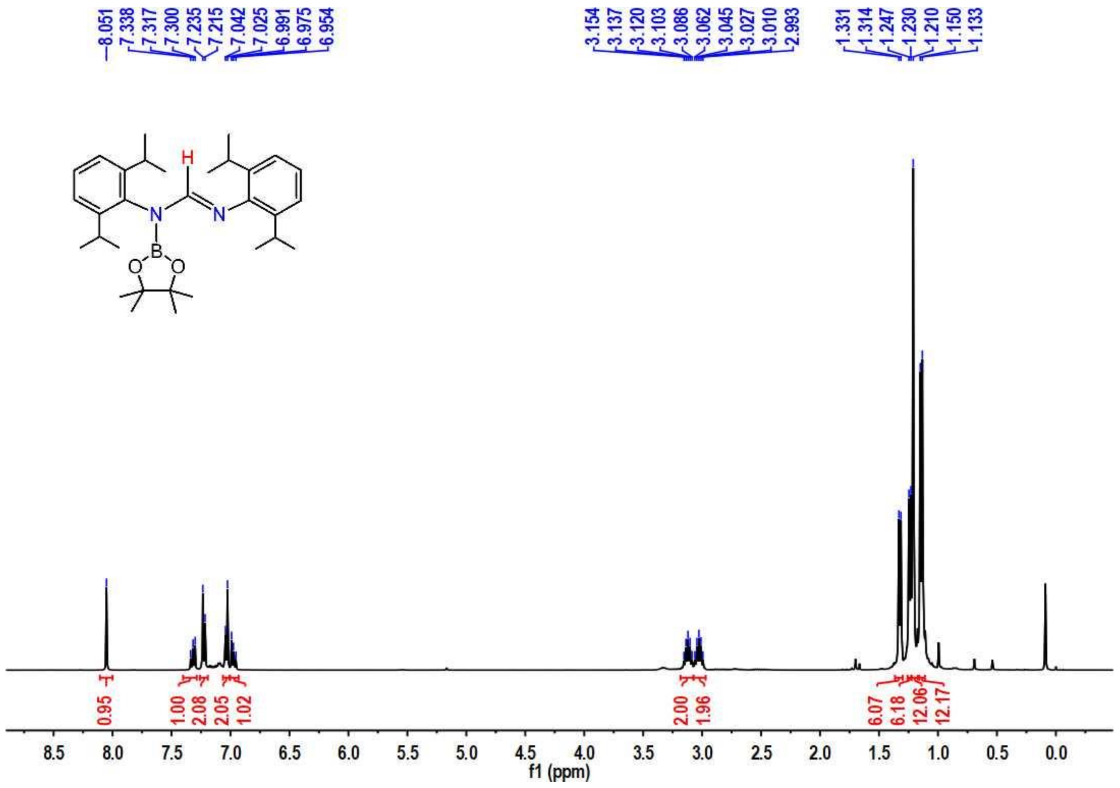

Figure S17. ^{1}H NMR spectrum (400 MHz, 298 K, $CDCl_3$) of d.

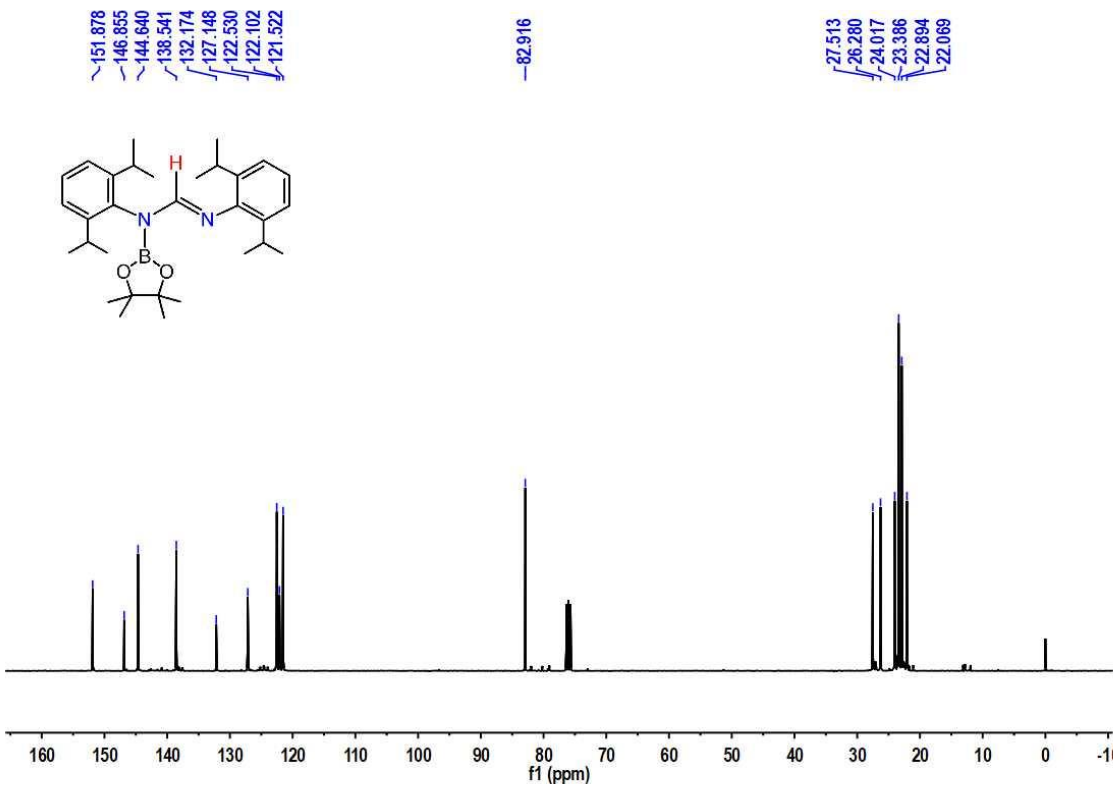

Figure S18. **^{13}C NMR spectrum (100 MHz, 298 K, $CDCl_3$) of d.**

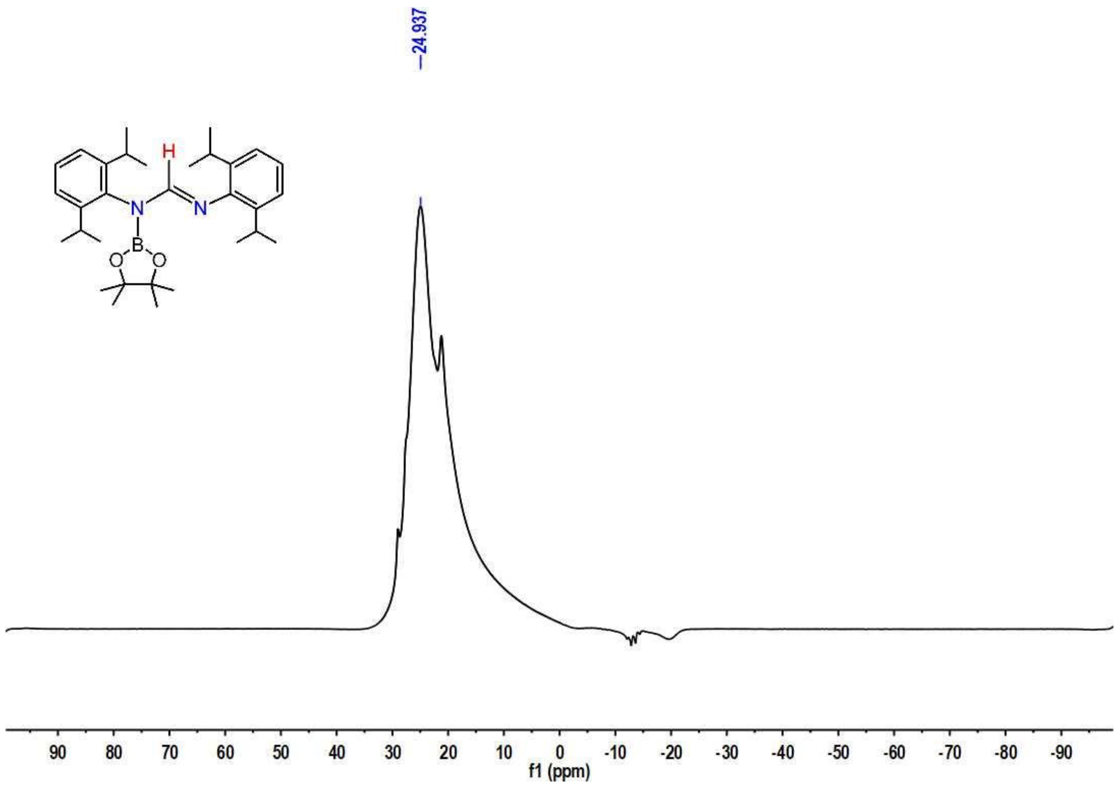

Figure S19. **^{11}B NMR spectrum (128 MHz, 298 K, $CDCl_3$) of d.**

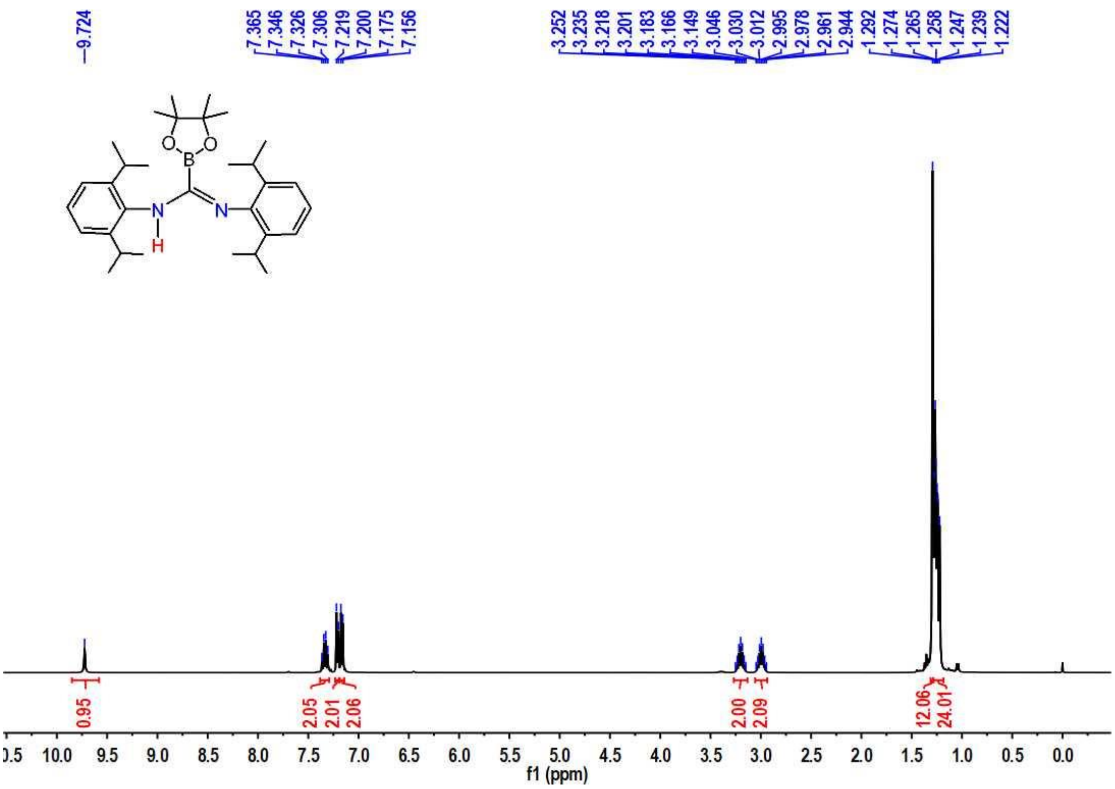

Figure S20.^{1}H NMR spectrum (400 MHz, 298 K, $CDCl_3$) of e.

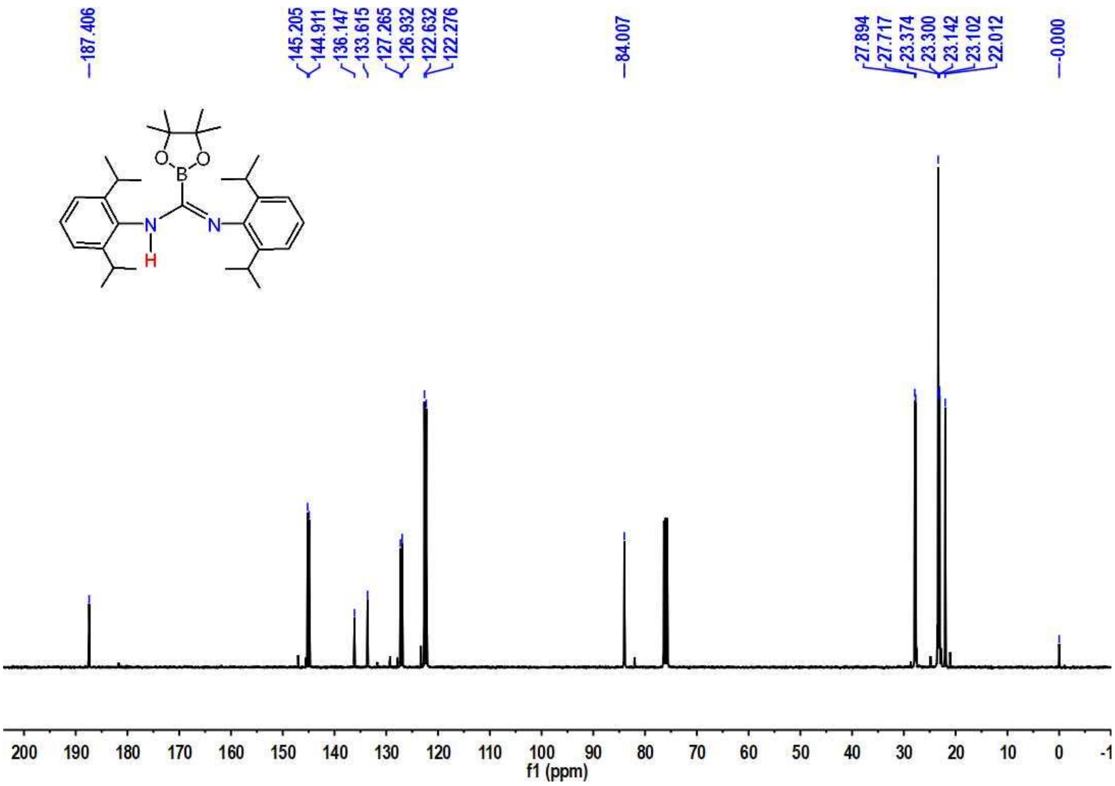

Figure S21. ^{13}C NMR spectrum (100 MHz, 298 K, $CDCl_3$) of e.

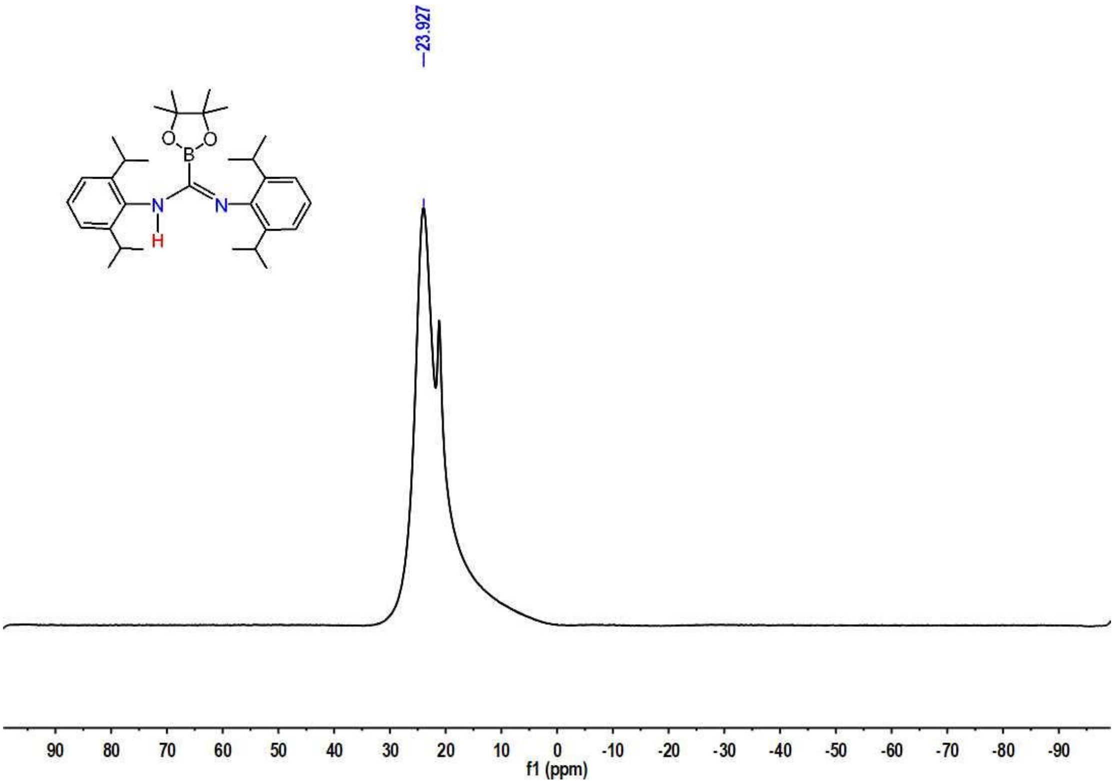

Figure S22. ^{11}**B NMR spectrum (128 MHz, 298 K, CDCl**$_3$**) of e.**

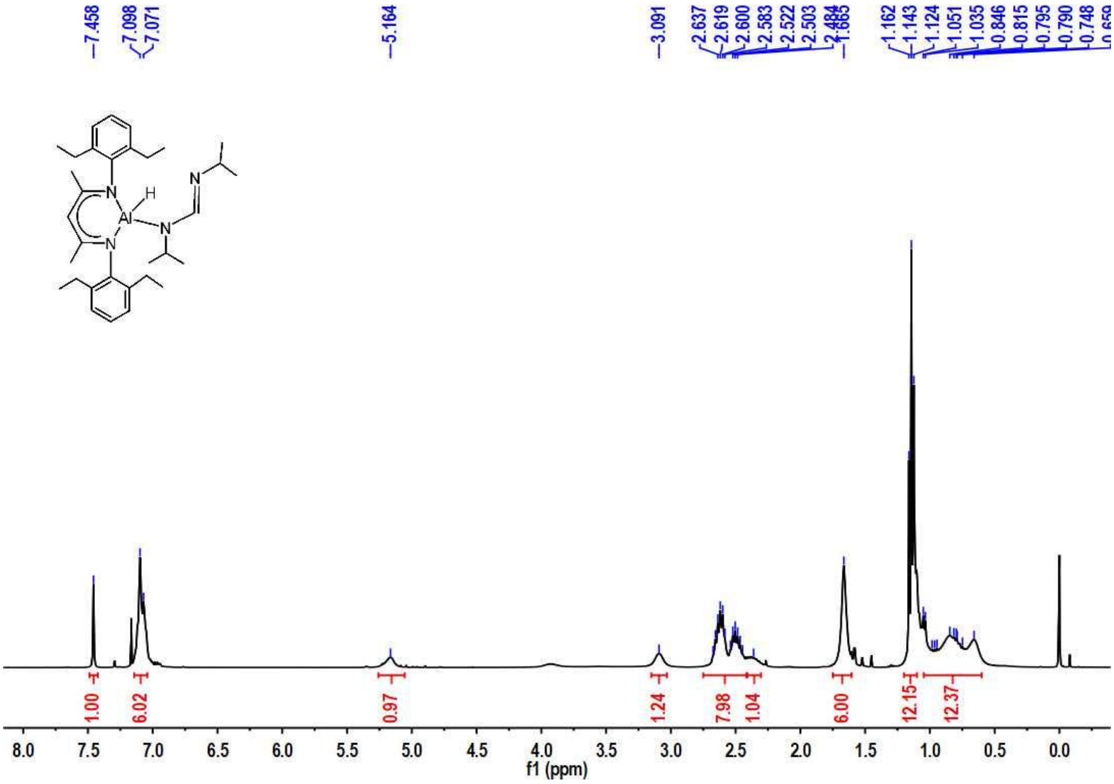

Figure S23.^{1}**H NMR spectrum (400 MHz, 298 K, CDCl**$_3$**) of complex 6.**

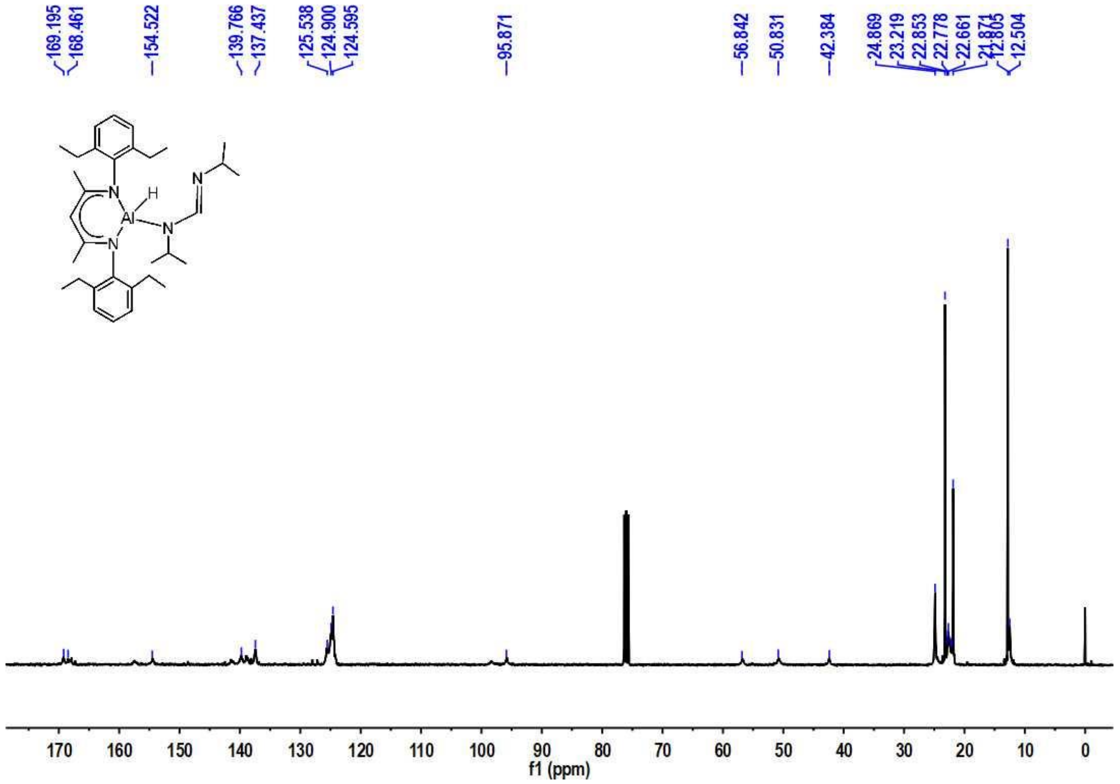

Figure S24.[13]**C NMR spectrum (100 MHz, 298 K, $CDCl_3$) of complex 6.**

Table S1: Crystal structure data and refinement details for complexes 2, 3 and 6.

Compound	**2**	**3**	**6**
CCDC	1893454	1893461	1893582
Empirical formula	$C_{20}H_{30}AlFN_2$	$C_{36}H_{47}AlN_4$	$C_{32}H_{49}AlN_4$
Formula weight	344.44	562.75	516.73
Temperature (K)	296(2)	296(2)	296(2)
Crystal system	monoclinic	triclinic	orthorhombic
Space group	$P2_1/c$	$P\bar{1}$	*Pnma*
a (Å)	11.9377(9)	11.4437(18)	9.0479(6)
b (Å)	10.2502(8)	12.794(2)	20.4254(13)
c (Å)	16.7653(13)	13.599(2)	17.0298(11)
α (deg)	90.00	95.781(5)	90
β (deg)	90.930(2)	97.628(5)	90
γ (deg)	90.00	99.042(5)	90
V (Å^3)	2051.2(3)	1933.8(5)	3147.2(4)
Z	4	2	4
ρ_c (g/cm^3)	1.115	0.966	1.091
μ/mm^{-1}	0.111	0.078	0.090
Crystal size(mm)	0.2×0.15×0.1	0.1×0.05×0.02	0.3×0.15×0.1
Θ range (deg)	1.706-28.330	1.523-24.146	2.39-24.28
Reflections collected	24713	16955	27986
$R_{(int)}$	0.0231	0.0455	0.0497
Data/restraints/parameters	5100/2/225	6128/15/443	2732/7/180
F(000)	744	608	1128
$R1^a$, $wR2^b$($I > 2\sigma(I)$)	0.0586, 0.1511	0.0795, 0.1411	0.0631, 0.1736
$R1^a$, $wR2^b$(all data)	0.0680, 0.1593	0.0536, 0.1322	0.0749, 0.1835

References

[1] C. Cui, H. W. Roesky, H. Hao, H. G. Schmidt, M. Noltemeyer, *Angew. Chem. Int. Ed. Engl.* **2000**, *112*, 1815−1817.

[2] W. Wang, Z. Yang, X. Ma, H. W. Roesky, Y. Ju, P. Hao, *Z. Anorg. Allg. Chem.* **2015**, *641*, 684-687.

[3] a) M. Bouyahyi, R. Duchateau, *Macromolecules* **2014**, *47*, 517-524; bBaixin Qian, Donald L. Ward, M. R. Smith*, *Organometallics* **1998**, *17* 3070–3076.

[4] a) H. Gao, Z. Ke, L. Pei, K. Song, Q. Wu, *Polymer* **2007**, *48*, 7249-7254; b) Q. Su, W. Gao, Q.-L. Wu, L. Ye, G.-H. Li, Y. Mu, *Eur. J. Inorg. Chem.* **2007**, *2007*, 4168-4175.

[5] T. W. Myers, N. Kazem, S. Stoll, R. D. Britt, M. Shanmugam, L. A. Berben, *J. Am. Chem. Soc.* **2011**, *133*, 8662-8672;

[6] a) M. Tian, J. Li, S. Zhang, L. Guo, X. He, D. Kong, H. Zhang, Z. Liu, *Chem. Commun.* **2017**, *53*, 12810-12813; b) J. Li, L. Guo, Z. Tian, M. Tian, S. Zhang, K. Xu, Y. Qian, Z. Liu, *Dalton Trans.* **2017**, *46*, 15520-15534; c) M. Belkhiria, A. Mechria, S. Dridi, T. F. C. Cruz, C. S. B. Gomes, P. T. Gomes, M. Msaddek, *J. Mol. Struct.* **2018**, *1171*, 827-833; d) C. Bianchini, H. Man Lee, G. Mantovani, A. Meli, W. Oberhauser, *New J. Chem.* **2002**, *26*, 387-397.

[7] a) C. Weetman, M. S. Hill, M. F. Mahon, *Chem. - Eur. J.* **2016**, *22*, 7158-7162; b) H. Liu, K. Kulbitski, M. Tamm, M. S. Eisen, *Chem. - Eur. J.* **2018**, *24*, 5738-5742.
